# Nanocomposites for Water Treatment, Photocatalysis, and Challenges: A Systematic Review

**DOI:** 10.1002/gch2.202500217

**Published:** 2025-08-26

**Authors:** Swellam W. Sharshir, Sabbah Ataya, Heba G. El‐Attar, Lotfy A. Lotfy, Ahmed A. El‐Naggar, Ahmed El‐Harairy, Mohamed M. Kedra, Abdulrhman M. Alaraj, Ahmed Sowayan, Rashid Khan, Mahmoud Abdelfatah, Abdelhamid El‐Shaer

**Affiliations:** ^1^ Mechanical Engineering Department Faculty of Engineering Kafrelsheikh University Kafrelsheikh 33516 Egypt; ^2^ Department of Mechanical Engineering College of Engineering Imam Mohammad Ibn Saud Islamic University (IMSIU) Riyadh Saudi Arabia; ^3^ Chemistry Department Faculty of Science Tanta University Tanta 31527 Egypt; ^4^ Physics Department, Nanoscience and Technology program Faculty of Science Kafrelsheikh University Kafrelsheikh 33516 Egypt; ^5^ Department of Chemical and Biomolecular Engineering College of Engineering University of Nebraska‐Lincoln Lincoln NE 68588 USA; ^6^ Environmental, Energy, and Green Chemistry Laboratory Faculty of Agriculture Damietta University Damietta 34517 Egypt

**Keywords:** bibliometric analysis, nanocomposites, photocatalysis, review, water treatment

## Abstract

The finite supply of water on this planet led researchers to investigate nanocomposites, which are unique compounds with great performance and many applications. Many researchers are now interested in photocatalytic degradation methods due to their ability to facilitate both spontaneous and non‐spontaneous reactions using light energy. The review's objective is to explain what nanocomposites mean, their types, various preparation procedures, and various characterisation approaches, and to employ nanocomposites in catalytic applications for wastewater treatment. It also seeks to compile some of the research on this topic. Through bibliometric analysis, the lineage and the extent to which countries are interested in publishing research on this issue in various methods of narration are illustrated. Nanocomposites can be used as catalysts to remove more than 90% of Cr (VI) after 120 min, phosphate (99.77%), ammonia (65.65%), Nitrite (99.98%) and remove several dyes such as Direct Blue 14 (94.57%), Congo Red (90.23%), Sunset Yellow (83.56%), brilliant cresol blue (BCB) (98.80%), neutral red (NR) (98.33%), methylene blue (MB) (99.6%) and more. Finally, challenges faced by nanocomposites in wastewater treatment are analyzed and summarized.

## Graphical abstract

A nanocomposite synthesized via sol‐gel, chemical vapor deposition, or in‐situ polymerization effectively removes dyes and heavy metals from water, yielding purified water. Additionally, it exhibits high photocatalytic degradation efficiency under visible or UV light. This demonstrates how nanocomposites contribute to solving environmental challenges in general, particularly water‐related issues, by providing efficient and sustainable solutions for water purification.

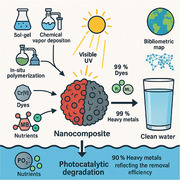

## 1 Introduction

Nanomaterials are often defined in terms of their size, with particles and materials with a size range of 1 to 100 nm. However, there is no accepted standard for this definition, as different organizations and governing bodies have different interpretations of what constitutes a nanomaterial. The Environmental Protection Agency suggests that nanomaterials possess features that set them apart from their traditional counterparts.^[^ ^1^ ^]^ Similarly, nanomaterials are defined by the Food and Drug Administration of the United States as substances between 1 and 100 nm at least in 1D. Additionally, nanomaterials are defined by the International Organization for Standardization Substances with internal nanoscale surface features.^[^ ^2^, ^3^, ^4^ ^]^ Examples of nanomaterials include nanoplates, nanowires, quantum dots, and nanofibers.^[^ ^5^, ^6^ ^]^ The key difference between nanomaterials and bulk materials is their size, as nanomaterials have a very small size compared to bulk materials.^[^ ^7^, ^8^ ^]^ As a result, nanomaterials and bulk materials have different chemical, physical properties, and applications. Nanomaterials are used in various fields and have been acknowledged for their potential in medical, energy, industrial, wastewater, and other sectors.^[^ ^9^, ^10^, ^11^, ^12^ ^.]^

Nanocomposites are advanced materials made up of two or more components; one must be on the nanometre scale (measuring less than 100 nanometres). They are often composed of metallic, polymeric, or non‐metallic materials, and the special combination of these components can result in enhanced properties.^[^ ^12^, ^13^ ^]^ Nanocomposites have a significant surface‐to‐volume ratio, which is one of their key benefits. This feature improved mechanical characteristics, including scratch resistance and ductility without a loss of strength, and improved optical properties, such as light transmission.^[^ ^14^ ^]^ Building on the fundamentals of nanocomposites, will now explore their synthesis and characterization methods, crucial steps in tailoring their properties for effective application in wastewater treatment.

### 1.1 Nanocomposites

Nanocomposites are advanced materials made by combining two or more distinct components, where at least one has nanoscale dimensions. These materials offer superior mechanical, thermal, and functional properties compared to traditional composites or monolithic materials, making them a strong alternative for a wide range of applications.^[^ ^15^ ^]^ The matrix, usually present in greater quantity, holds the structure together, while nanoscale fillers, such as carbon nanotubes or clay, act as reinforcements to enhance the composite's performance. Nanocomposites can take various forms, including 1, 2, and 3D structures, and can be engineered with multifunctional properties due to the diversity in their components’ shapes and compositions. A major advantage of nanocomposites lies in their significantly larger interfacial area between the matrix and the nanoscale reinforcement, which contributes to their enhanced behavior. Since the introduction of carbon nanotubes in 1991, research on nanocomposites has rapidly expanded, with growing interest from both academic and industrial fields, particularly in polymer/clay systems, due to their improved strength, thermal stability, and versatility.^[^ ^16^ ^]^

### 1.2 Types of Nanocomposites

**Figure 1:**
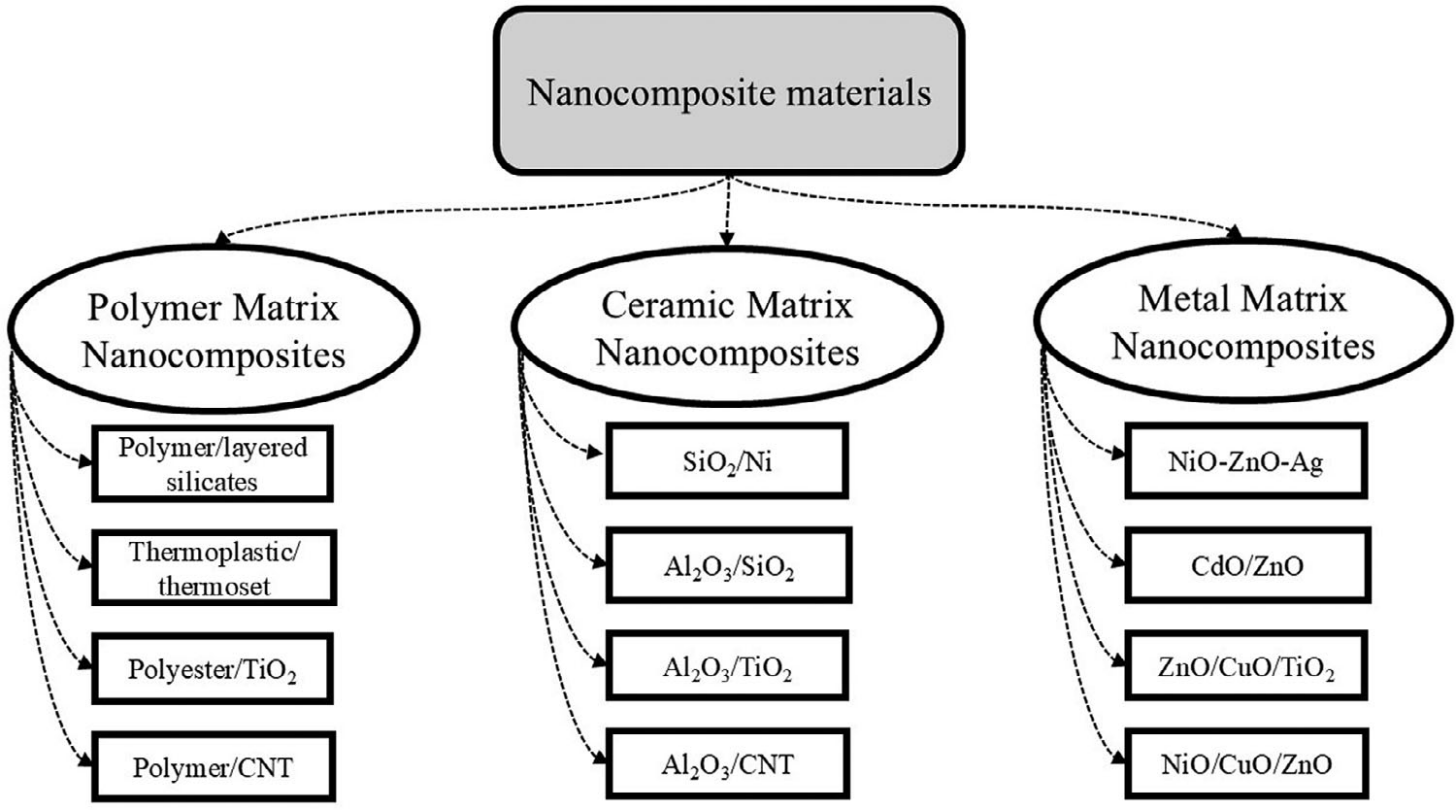
Types of Nanocomposites and classification of three types of Nanocomposites.

Nanocomposites are broadly classified into three main categories based on the type of matrix material, as shown in **Figure** **1**. They contain: Polymer Matrix Nanocomposites (PMNCs), Ceramic Matrix Nanocomposites (CMNCs), and Metal Matrix Nanocomposites (MMNCs). This classification, supported by researchers like Lateef, Nazir, and Parameswaranpillai,^[^ ^17^ ^]^ reflects how the matrix material significantly influences the composite's properties and potential applications. PMNCs are widely used due to their lightweight nature, flexibility, and ease of processing, making them suitable for packaging, automotive, and biomedical uses. CMNCs, which use ceramics as the matrix, offer high thermal stability, hardness, and resistance to wear, making them ideal for high‐temperature and structural applications. MMNCs, composed of metal matrices, provide excellent mechanical strength, electrical conductivity, and durability, which are essential in aerospace, electronics, and automotive industries. This matrix‐based classification helps guide material selection and design for specific engineering and industrial needs.^[^ ^18^ ^]^

#### 1.2.1 Polymer Nanocomposites (PNCs)

Polymer nanocomposites (PNCs) represent an innovative class of hybrid materials formed by embedding nanoscale fillers into a polymer matrix to significantly enhance the base polymer's properties. These nanofillers range from 1D structures like nanotubes and nanofibers to 2D materials like clay and 3D spherical particles, which interact with the polymer matrix at the molecular level, resulting in improved mechanical, thermal, and electrical properties.^[^ ^19^ ^]^ Polymers are widely chosen as matrices due to their lightweight nature, high durability, ductility, corrosion resistance, ease of processing, and cost‐effectiveness. Despite their benefits, polymers inherently have limited structural and functional properties compared to ceramics or metals. The key to achieving high‐performance nanocomposites lies in the uniform dispersion and distribution of nanofillers; poor dispersion can lead to agglomeration, acting as defects and diminishing the expected enhancements. Ideally, polymer matrix nanocomposites feature well‐separated nanoscale particles dispersed uniformly throughout the matrix, although in practical scenarios, dispersion remains a challenge. Functional PNCs are now enabling advancements in fields such as micro‐optics, electronics, energy storage, and conversion.^[^ ^16^ ^]^ Their performance is closely tied to filler concentration, rheological behavior, and interfacial interactions, which must be carefully evaluated prior to composite processing. Various types of polymers, such as thermoplastics, thermosets, elastomers, and biodegradable options, serve as matrices, while methods like the sol–gel process allow nanoparticles to be integrated at near‐molecular scales. Over the past decade, this field has garnered significant global attention for its potential in creating advanced materials with tailored functionalities.^[^ ^20^ ^]^

#### 1.2.2 Ceramic Matrix Nanocomposites (CMNCs)

Ceramic matrix nanocomposites (CMNCs), especially those based on aluminum oxide (Al_2_O_3_) reinforced with silicon carbide (SiC), have shown remarkable potential in improving the performance of traditional ceramics. Studies by researchers like Nihara and Gurnani have confirmed that incorporating a small amount, ≈10% by volume of appropriately sized SiC particles into an Al_2_O_3_ matrix significantly enhances the mechanical properties of the composite, particularly its toughness and resistance to sudden failure.^[^ ^21^, ^22^ ^]^ This improvement is largely attributed to the crack‐bridging effect provided by the nanoscale reinforcements, such as nanofibers or particles, which prevent crack propagation and increase fracture resistance. Unlike monolithic ceramics, which are prone to brittle fracture, these nanocomposites exhibit superior failure characteristics and toughness. Various fabrication methods are used to create CMNCs, including traditional powder processing, vapor deposition techniques (CVD and PVD), spray pyrolysis, polymer precursor routes, and chemical approaches like sol–gel synthesis, colloidal processing, precipitation, and template‐assisted synthesis. Among these, the sol–gel method is particularly notable for its ability to finely control the structure and chemical properties of the final composite by adjusting parameters such as solvent type, reaction time, pH, water‐to‐metal ratio, and precursor materials.^[^ ^23^ ^]^ Common CMNC systems include Al_2_O_3_/SiO_2_, SiO_2_/Ni, Al_2_O_3_/TiO_2_, and notably Al_2_O_3_/SiC. In addition, carbon nanotubes (CNTs) have become widely used nanofillers due to their exceptional strength and conductivity, with composites such as Al_2_O_3_/CNT, MgAl_2_O_4_/CNT, and MgO/CNT emerging as promising materials for advanced structural applications.

#### 1.2.3 Metal Matrix Nanocomposites (CMNCs)

Metal matrix composites (MMCs) are advanced materials composed of two or more distinct phases—typically a metallic matrix and a reinforcing phase such as fibers or particles, that are combined to produce enhanced properties not achievable by either component alone. The reinforcement can be fibrous or particulate and is uniformly distributed within the metal to improve mechanical behavior. A common example includes aluminum matrices reinforced with continuous aluminum oxide (Al_2_O_3_) fibers used in power transmission lines for their high strength‐to‐weight ratio.^[^ ^18^ ^]^ Other well‐known MMCs include cobalt (Co) with tungsten carbide (WC) used in cutting tools and drilling inserts due to their hardness and wear resistance, and aluminum reinforced with silicon carbide (SiC) particles, widely applied in automotive parts, aerospace components, and systems requiring effective thermal management. When these composites are developed at the nanoscale, which leads to forming metal matrix nanocomposites (MMNCs), they combine the toughness and ductility of metals with the high stiffness and strength of ceramics. These nanoscale reinforcements significantly improve the material's performance under extreme conditions, including high temperatures, shear stress, and compression. Due to their excellent mechanical and thermal properties, MMNCs are considered highly promising for use in high‐performance structural applications across a broad range of sectors such as aerospace, automotive engineering, and heavy industrial manufacturing.^[^ ^24^ ^]^

### 1.3 Preparation of Nanocomposites

**Figure 2:**
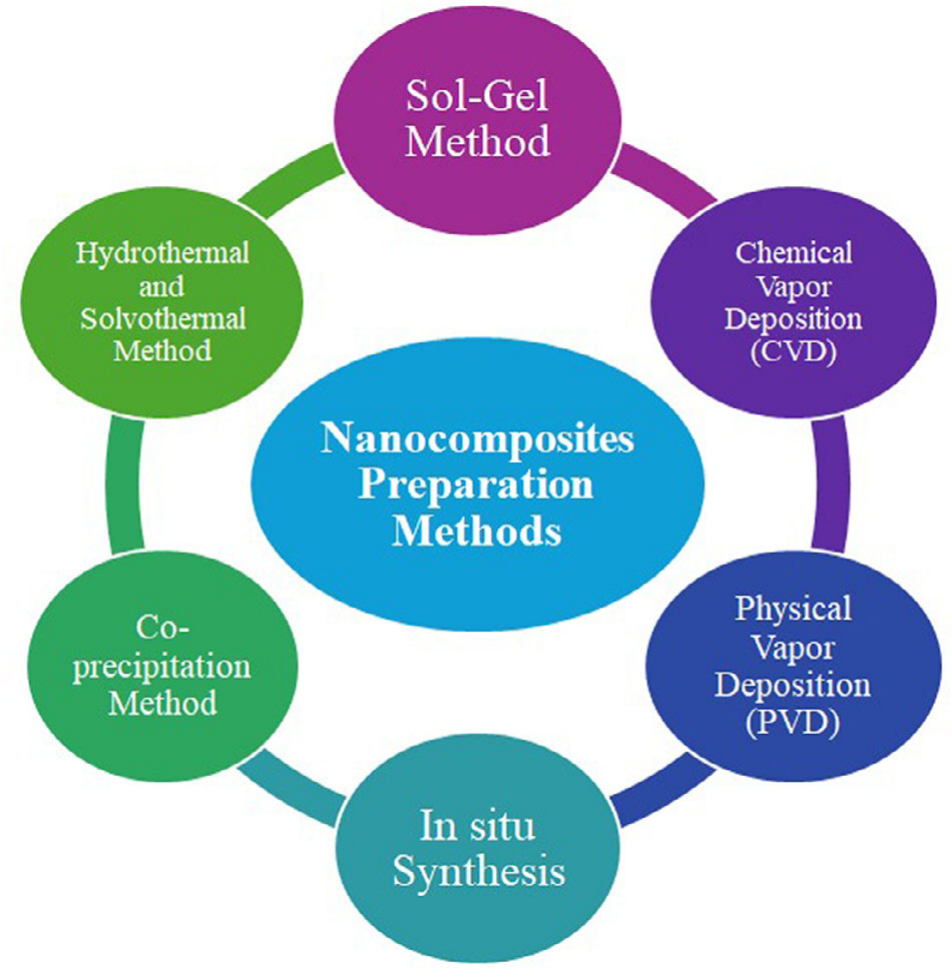
Demonstrates the prevailing methods for preparing nanocomposites.

Synthesis methods are mainly categorized into top‐down (physical) and bottom‐up (wet chemical) approaches. Top‐down methods can produce large quantities but struggle with size uniformity, while bottom‐up methods offer better control over size and shape, allowing for various nanostructures like nanorods and nanotubes. The choice of method depends on the desired characteristics of the final nanocomposite. Numerous methods exist for synthesising nanocomposites, and each comes with its unique advantages and disadvantages as shown in **Figure** **2**.

#### 1.3.1 Chemical Vapor Deposition

Chemical vapor deposition is a method of producing high‐performance solid materials and high‐quality. The semiconductor industry frequently uses this technique to create thin films. It involves putting the substrate in contact with volatile precursors, and the desired deposit is formed as a result of reaction and decomposition. By‐products, such as vapours, are then removed through a gas flow.^[^ ^25^ ^]^

Tian Zhang et al. used chemical vapour deposition (CVD) to synthesize Fe_3_O_4_/CNTs nanocomposites with Fe_2_O_3_/NaCl nanoparticles as catalysts. The optimal conditions were 0.5 weight percent of Fe_2_O_3_/NaCl catalyst, a 1:4:2 mole ratio of ethyl acetate to water to oxygen, and a growth time of 3 h. In the non‐enzymatic electrochemical detection of H_2_O_2_, the generated Fe_3_O_4_/CNTs nanocomposites (i.e., 0.5Fe‐4H_2_O‐3) showed the best performance, improving the electrochemical characteristics of the sensor and the interaction between Fe_3_O_4_ and CNTs.^[^ ^26^ ^]^

#### 1.3.2 Physical Vapor Deposition

Physical vapour deposition is a vacuum deposition method used to create thin films and coatings. The substance must pass through three phases: condensed, vapour, and thin film. Sputtering and evaporation are two of the most used techniques for physical vapour deposition. Three processes make up the process: sputtering/evaporating various components to create a vapour phase, supersaturating the vapour phase in an inert atmosphere, and thermally consolidating the nanocomposite.^[^ ^27^, ^28^ ^]^

A PVD technique called liquid plasma‐assisted particle deposition sintering (LPDS) was used in the study by Xinrui Zhao et al. to create TiO_2_‐hBN nanocomposite coatings. With the help of this procedure, hBN was uniformly deposited on Ti6Al4V, resulting in an 86.7% coating density. The coating's potential for anti‐friction and corrosion resistance applications was highlighted by the friction coefficient, which dropped dramatically from 0.54 to 0.28 and remained stable after 2000 sliding cycles.^[^ ^29^ ^]^

#### 1.3.3 Sol–Gel Method

Ceramic nanocomposites are frequently created using the sol–gel method. In this process, a molecular precursor that is either organic or inorganic, which has been dissolved in an organic medium, undergoes hydrolysis processes, which leads to the production of polymers in 3D with metal‐oxygen links. The substance is heated to consolidate it after being dried to form a solid.^[^ ^30^ ^]^

In Saeid Taghavi Fardood's work, MgFe_2_O_4_@CeO_2_ superparamagnetic nanocomposites were prepared using the sol–gel process, a method that involves controlled preparation of the nanocomposite through the synthesis of a gel precursor and subsequent thermal treatment to achieve the final product. The process is capable of offering the best control over the composition and morphology of the nanocomposites, therefore achieving superparamagnetic nanocomposites with desirable properties.^[^ ^31^ ^]^

Syed Salman Shafqat et al.’s study describes the sol–gel synthesis of multifunctional nitrophenylfurfural grafted silica nanoparticles (NPF–SiNPs). Amino‐functionalized silica nanoparticles (AFSi‐NPs) were initially developed by functionalizing silica nanoparticles with amino groups. The NPF–SiNPs were then prepared by post‐grafting nitrophenylfurfural derivatives (para, ortho, and meta) onto the surface of AFSi‐NPs. The resultant NPF–SiNPs showed a notable surface area of 80 m^2^ g^−1^, especially those modified with p‐nitrophenylfurfural. When the NPF–SiNPs were tested for their capacity to adsorb tartrazine (TTZ) dye from wastewater, p‐NPF–SiNPs demonstrated greater activity. The silica nanoparticles' functionalisation and preparation technique were crucial in increasing the material's adsorption efficiency and capacity.^[^ ^32^ ^]^

The sol–gel method is a low‐temperature, versatile technique for synthesizing nanocomposites using metal alkoxides or organometallic precursors. It offers advantages like high chemical homogeneity, precise stoichiometry control, high purity, and the ability to form 3D metal‐oxygen networks. It is suitable for producing single or multi‐phase composites, especially from liquids or viscous fluids. However, it has limitations such as significant shrinkage during drying and lower porosity compared to conventional mixing methods. Despite this, it remains a popular and effective method for creating high‐performance nanocomposite materials.^[^ ^33^ ^]^

#### 1.3.4 In Situ Synthesis

In situ synthesis is a technique of producing nanocomposites, which involves the combination of precursors, including metal ions for nanomaterials or monomers for polymers. There are three variations of this procedure based on the components and manufacturing techniques. The first type involves combining polymer with nanoparticle precursors, then applying a gas or liquid containing S_2_, OH, and Se_2_, making it possible to create the in situ nanoparticles required. The second type uses polymer and nanomaterial precursors only, where the goal is to prevent aggregation and enhance the interfacial interaction between the polymer and the nanomaterial by dispersing nanoparticles in the precursors or monomers of the polymer. In order to create the nanocomposite, the entire mixture is then polymerized. The third type involves using precursors of both polymers and nanomaterials, which produce nanoparticles and polymers simultaneously.^[^ ^34^, ^35^ ^]^ In situ synthesis is a beneficial method for creating nanocomposites due to its ability to tailor physical properties. It is an effective way of producing nanomaterials and polymers for many applications.^[^ ^36^ ^]^

Ag nanoparticles (Ag NPs) were synthesized in situ on the surface of nano silica (SiO_2_) using gamma radiation in a research reactor by M. Bagherzadeh et al. The samples were 25 kGy irradiated in a special radiation cell that allowed penetration of gamma radiation to the samples without neutron exposure; thus, radioactivity was avoided. This in situ strategy enabled the uniform deposition of Ag NPs on the SiO_2_ surface, with the nanocomposite displaying fine dispersion of Ag NPs with a size of 100 ± 4 nm. The Ag NPs/SiO_2_ nanocomposite showed potential for catalysis and the reduction decolourisation of dyes like methylene blue (MB), Congo red (CR), and methyl orange (MO). This indicates the effectiveness of the in situ synthesis process in the production of nanocomposites that can be utilized for environmental cleanup and pollution control.^[^ ^37^ ^]^

#### 1.3.5 Co‐Precipitation Method

The co‐precipitation method is a widely used chemical approach for synthesizing nanocomposites, where metal cations are simultaneously precipitated from a common solution, typically as hydroxides, carbonates, oxalates, or citrates. After precipitation, the resulting compounds are subjected to calcination at relatively low temperatures, leading to the formation of fine nano powders with smaller particle sizes. This method begins with simple mixing of reactants, often in a beaker, but achieving high‐quality nanomaterials requires precise control over several key parameters, such as reactant concentration, order and timing of addition, solution pH, process temperature, and the use of surfactants. Once the solution reaches supersaturation, spontaneous nucleation occurs, followed by a controlled growth phase that shapes the final nanostructures.^[^ ^38^ ^]^

Despite its simplicity and cost‐effectiveness, co‐precipitation presents certain challenges. One major issue is contamination from reaction byproducts, which can affect the purity of the final material. In addition, minor variations in working conditions, such as stirring speed, light exposure, vibration, or even the cleanliness of the equipment, can significantly influence the final product's morphology, structure, and composition. Furthermore, this method may not be suitable for producing high‐purity phases with exact stoichiometry, especially if the involved reactants differ in solubility. The choice of precipitating agent (e.g., NH_4_OH, NH_4_HCO_3_, or (NH_3_)_2_CO_3_) and the drying technique also play critical roles in determining the final characteristics of the nanocomposite.^[^ ^39^ ^]^

#### 1.3.6 Hydrothermal Methods

The hydrothermal method is a versatile technique for synthesizing nanocomposites through chemical reactions carried out in a sealed vessel containing a solvent, either aqueous or non‐aqueous, at elevated temperatures and pressures above atmospheric levels.^[^ ^40^ ^]^ This method enables controlled crystal growth and allows tuning of particle size, shape, and structure by adjusting parameters such as temperature, pressure, reaction time, and the use of additives like surfactants, capping agents, or mineralizers.^[^ ^41^ ^]^ To enhance the quality and stability of the final product, modern approaches often combine hydrothermal synthesis with other techniques such as microwave‐assisted heating^[^ ^42^ ^]^ or the sol–gel process.^[^ ^43^ ^]^ These combinations help tailor the physicochemical properties of the nanocomposites and can lead to the formation of single‐phase, highly stable materials. The advantages of the hydrothermal method for the synthesis of nanocomposites include precise control over morphology, the ability to synthesize complex structures at relatively low temperatures, and the production of high‐purity and well‐crystallized materials. The limitation involves the need for specialized high‐pressure equipment, longer reaction times, and difficulty in scaling up the process for industrial applications.

### 1.4 Characterization of Nanocomposites

**Figure 3:**
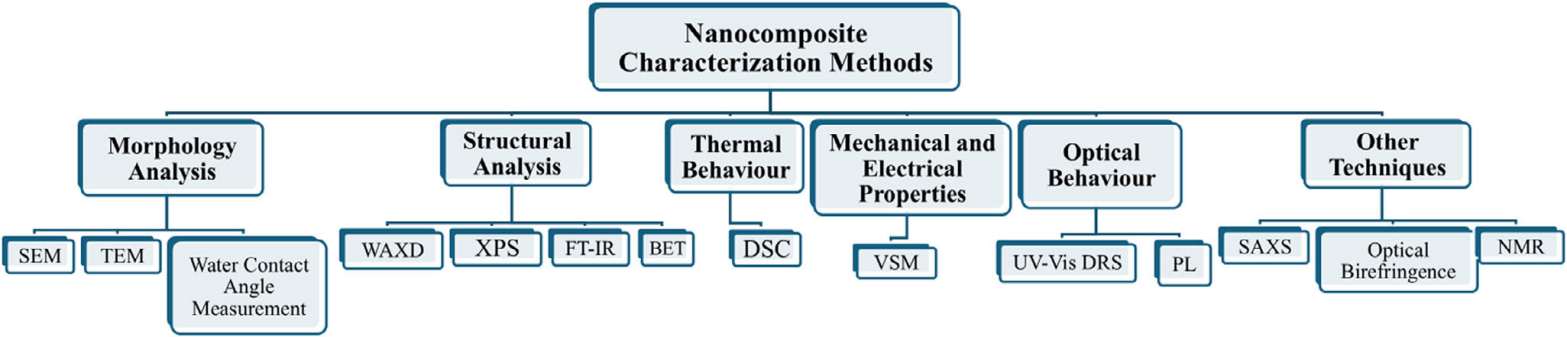
Schematic Representation of Nanocomposite Characterization Methods.

Modern nanocomposites are produced by fusing the distinct qualities of nanoparticles and polymers. These composites allow for the fabrication of high‐performance materials with improved thermal, electrical, mechanical, and barrier properties. These materials are characterized by using different techniques as shown in **Figure** **3**. To research the morphology of the surface and macroscale morphology of nanocomposites, researchers use a variety of techniques such as transmission electron microscopy (TEM), scanning electron microscopy (SEM), small angle X‐ray scattering (SAXS), differential scanning calorimetry (DSC), wide angle X‐ray diffraction (WAXD), Fourier transform infrared (FT‐IR), optical birefringence, nuclear magnetic resonance (NMR), X‐ray photoelectron spectroscopy (XPS), vibrating sample magnetometer (VSM), Brunauer–Emmett–Teller (BET) and water contact angle measurement.^[^ ^44^, ^45^, ^46^ ^]^ For morphological analysis, SEM is the simplest and most widely utilized method,^[^ ^47^, ^48^ ^]^ while for qualitative investigation and structural defect analysis, TEM is frequently used.^[^ ^44^, ^49^ ^]^ WAXD is an effective method for investigating the structure of nanocomposites by observing the shape, position, and intensity of basal reflections.^[^ ^50^, ^51^ ^]^ However, it provides little information about the spatial distribution or structural flaws of the nanocomposite. DSC, on the other hand, is used to analyse the thermal behavior of the nanocomposite.^[^ ^52^ ^]^

BET (method is commonly used to analyze the surface area and porosity of nanocomposites after synthesis. By measuring the amount of nitrogen gas adsorbed onto the material's surface, BET analysis provides valuable data on the specific surface area, which is crucial for applications in catalysis, adsorption, and energy storage. In nanocomposite synthesis, a high BET surface area typically indicates well‐dispersed nanofillers and enhanced interfacial interaction between the matrix and reinforcement. This characterization helps evaluate the effectiveness of the synthesis method and the suitability of the material for specific functional applications.^[^ ^31^ ^]^ UV–vis DRS (Diffuse Reflectance Spectroscopy) and Photoluminescence (PL) spectroscopy are essential techniques used to characterize the optical properties of nanocomposites after synthesis. UV–vis DRS helps determine the material's light absorption behavior and estimates the optical bandgap, which is crucial for applications in photocatalysis and optoelectronics. By analyzing the reflectance spectra, researchers can assess how nanocomposite components influence light interaction and electronic transitions.^[^ ^53^ ^]^ PL spectroscopy provides insights into the recombination behavior of photo‐generated charge carriers. A strong PL signal typically indicates high recombination rates, while reduced PL intensity suggests efficient charge separation, which is desirable in applications like solar cells and photocatalysis.^[^ ^54^ ^]^

Ala Manohar et al. Characterization of Mg_0.7_Ni_0.3_Fe_2_O_4_/CeO_2_/MgFe_2_O_4_ Nanocomposite Material using various techniques. The Mg_0.7_Ni_0.3_Fe_2_O_4_/CeO_2_/MgFe_2_O_4_ nanocomposite material was characterized using various techniques. XRD showed unambiguous phases and crystallite sizes of ≈15 nm (CeO_2_), 14 nm (MgFe_2_O_4_), and 12 nm (NiFe_2_O_4_). FE‐SEM and TEM showed spherical nanoparticles of diameter ≈14 nm. EDS and elemental mapping showed uniform distribution of Mg, Ni, Fe, Ce, and O. HR‐TEM identified lattice spacings, while FTIR measured metal‐oxygen vibrations. XPS revealed oxidation states of the elements, and EPR provided a g‐factor value of 2.29. VSM demonstrated a ferromagnetic nature (52.23 emu g^−1^). Electrochemical tests (CV, GCD, EIS) displayed a combination of capacitive and diffusion‐controlled charge storage with a specific capacitance of 167 Fg^−1^.^[^ ^55^ ^]^

Batool et al.^[^ ^56^ ^]^ investigated the synthesis of a BiSbS_3_@BiSbO_4_/CNH nanocomposite for wastewater treatment and electrochemical purposes. The authors employed several advanced characterization techniques to explore its properties. X‐ray diffraction (XRD) was used to determine the crystal structure and phase composition, which confirmed the existence of target phases. Scanning electron microscopy (SEM) and transmission electron microscopy (TEM) provided details about the morphology and nanostructured features of the material. Energy dispersive X‐ray (EDX) analysis confirmed the elemental composition and homogeneity of the nanocomposite. UV–vis spectroscopy assessed the low bandgap value of 2.64 eV of the nanocomposite responsible for its efficient photocatalytic degradation of pentachlorophenol (5‐CP) under visible light. These characterisation techniques provided a holistic view of the structure, optical, and morphological behavior of the material and were instrumental in optimising nanomaterials for environmental purposes.

Recent decades have seen an alarming rise in environmental problems, including resource depletion, climate change, and air and water pollution. Innovative solutions are urgently needed to reduce the adverse impact on our planet and provide a sustainable future as humankind struggles with these serious concerns. This effort has led to the development of nanotechnology as a ground‐breaking field with the potential to transform several industries, including environmental protection. The importance of nanocomposites in solving some of the most severe environmental problems confronting humanity today is explored in this introduction. One of the most significant and critical of these issues is the issue of wastewater and how to employ nanomaterials, especially nanocomposites.

This review will reveal the synthesis and characterization of nanocomposites by various techniques, including XRD, TEM, SEM, FT‐IR, photoluminescence spectroscopy, XPS, and UV–vis. The most recent approaches and solutions researchers have found for solving water problems (wastewater treatment). The bibliometric analysis illustrates the trends and the extent of global interest in publishing research on this topic. With a total of 876 research publications, India was one of the most published countries using nanocomposites for the treatment of water. As the year 2024 was the most published year for research papers in this field. Finally, challenges faced by nanocomposites in wastewater treatment are analysed and summarized.

## 2 Bibliometric Analysis of Nanocomposites

Bibliometric analysis is a quantitative technique for assessing and quantifying the influence of scholarly communication, including the impact of journals, authors, institutions, and scientific research publications. It involves analysing bibliographic data, which contains details on citations, co‐citations, publication frequency, and other relevant elements. Bibliometric analysis aims to learn more about scientific publications' patterns, trends, and influence within a certain subject or across disciplines. The data used for this analysis is often collected through trusted databases such as Scopus, Web of Science, Google Scholar, and PubMed, which provide comprehensive bibliographic and citation data to assess research trends and academic impact in various fields. In our work, Scopus will be our primary database for collecting the data and conducting the analysis.

Bibliometric analysis is frequently used in academia, research institutes, and funding agencies to distribute resources, determine the impact of research results, and choose collaborations and research directions. Although bibliometric analysis offers insightful quantitative information, it may not fully capture the qualitative components of research effect and relevance.

In **Figure** **4**a, the graph based on data from Scopus shows the number of documents published each year from 2015 to 2025 on a set of keywords (nanocomposite AND water‐treatment AND photocatalysis). Research interest in nanocomposites and water treatment through photocatalysis showed a significant increase until 2024, with publications peaking during this period. However, there is a noticeable decline in researcher interest after 2024, possibly reflecting stabilization in the field or a shift toward other topics or techniques. To revive interest, researchers could focus on improving the efficiency of nanomaterials for diverse water treatment applications, such as enhancing cost‐effectiveness and increasing the removal of various pollutants, which may help reignite attention and innovation in the field.

Figure 4Annual publication trends on (nanocomposite AND water‐treatment AND photocatalysis) in the period (2015–2025) based on Scopus data (a), Network visualization map for all keywords using VOS viewer (b), and concentration of research publications between 2015 and 2025 on all keywords (c).

**Figure:**
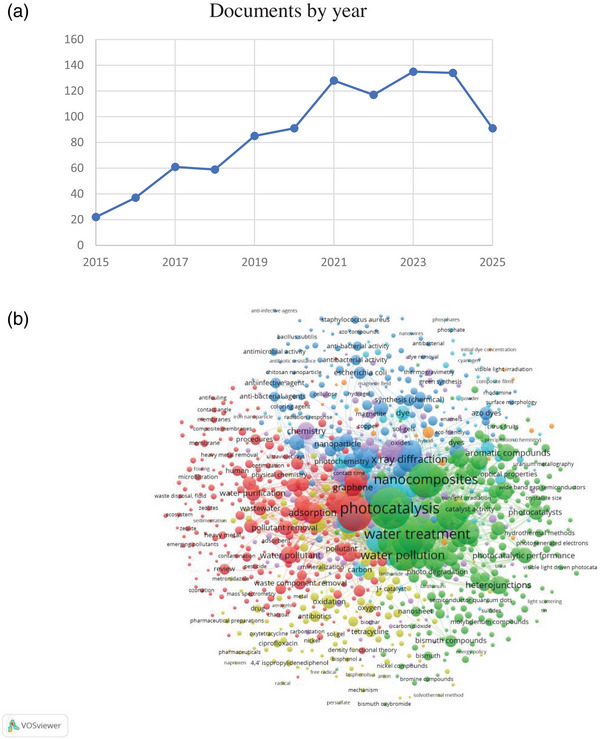

**Figure:**
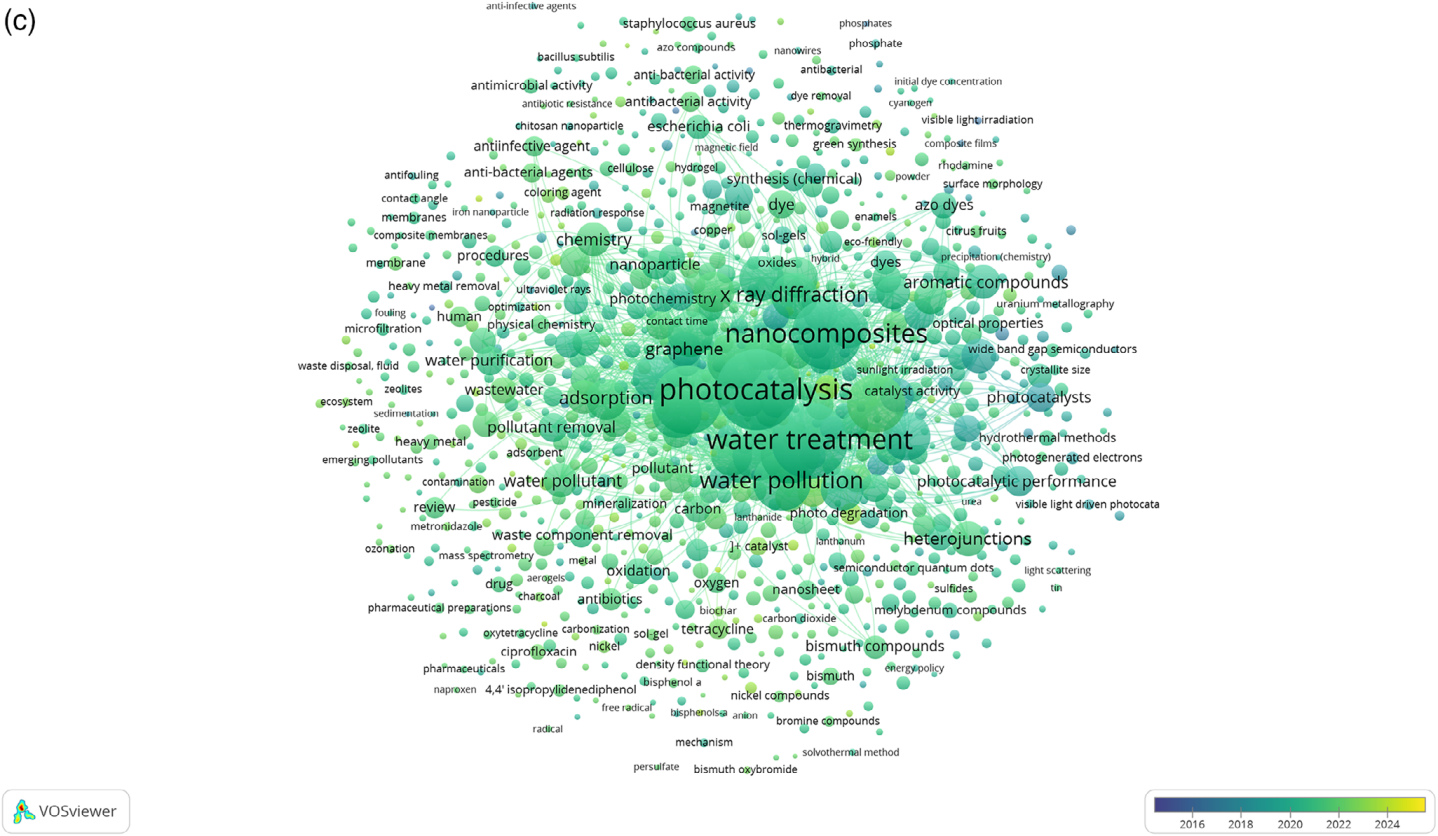

### 2.1 Bibliometric Maps

Using data from 876 articles, the VOSviewer software was used to create bibliometric maps that visualize relationships between countries and author keywords. Each link between objects, such as countries or keywords, is assigned an integer indicating its strength, with higher integers signifying stronger links.^[^ ^57^ ^]^ In the co‐authorship study, the strength of linkages between countries reflects the number of co‐authored papers. In contrast, the overall link strength indicates the total strength of a country's co‐authorship connections.^[^ ^58^ ^]^ Similarly, in the co‐occurrence analysis, the intensity of the association between author keywords reflects the frequency of publications that include both keywords.^[^ ^59^ ^]^

### 2.2 Analysis of Co‐Occurrence

The Scopus database was used to search for a set of keywords (nanocomposite AND water‐treatment AND photocatalysis) in the publication's title, abstract, and keywords. This co‐occurrence analysis of key research topics across 876 documents, the evaluation threshold was set to a minimum of 5 occurrences per term. Figure 4b shows the bibliometric analysis conducted using the VOS viewer, which clarify clusters of terms such as “photocatalysis,” “water treatment,” and “nanocomposites,” highlighting the strong connections within environmental and nanotechnology research. The size of the nodes indicates the frequency of terms, with larger nodes representing more commonly mentioned topics. The lines between the nodes signify the co‐occurrence of terms within the same documents, with thicker lines showing stronger relationships. Major themes identified include the intersection of photocatalysis with water treatment and pollution, as well as the increasing focus on nanomaterials and antibacterial activity, reflecting the emerging research trends in environmental sciences and nanotechnology. Figure 4c shows the network visualization of research related to these keywords from 2015 to 2024.

## 3 Using Various Nanocomposites in Water Treatment

For the specific purpose of removing Methyl Orange (MO) and Naproxen Sodium (NAP) from wastewater, a new nanocomposite called PANI/GO/MOF‐Fe_3_O_4_ was developed by Ejaz et al.^[^ ^60^ ^]^ The morphology and composition of the nanocomposite and its thermal stability were studied using several characterization techniques, which included FTIR, XRD, SEM, TGA, BET, and XPS. The adsorption capacities achieved were 239.78 mg g^−1^ for MO and 40.64 mg g^−1^ for NAP, which proves the effectiveness of the composite in pollutant removal.

Anirudhan et al.^[^ ^61^ ^]^ fabricated a polyacrylonitrile/organ bentonite composite with amidoxime functionality using an in situ polymerisation method. XRD, SEM, FT‐IR, surface area analyzer, thermogravimetry, and potentiometric analysis are used to describe the prepared composite. The research results can be used as a powerful sorbent to get rid of heavy metals found in aqueous solutions. The fluid's pH had an impact on sorption; removal was greatest at pH 6.0. Based on the tests, it was found that lower concentration levels (25.0 mg L^−1^) allow for ≈97–100% removal. Humic acid‐immobilized amine‐modified polyacrylamide/bentonite compound was prepared by Anirudhan et al.^[^ ^62^ ^]^ To determine the adsorbent's adsorptive properties and the surface features of the substance, several methods, including SEM, XRD, EDX, and FT‐IR. Some cationic dyes, such as CV, MB, and MG were adsorbed onto the prepared nanocomposite. The consumption of dyes was MG 199.4 mol g^−1^ (99.7%) was the greatest one, then MB 193.4 mol g^−1^ (96.7%), then CV 187.5 mol g^−1^ (93.8%) at 400 mol L^−1^ of dye concentration was used initially. The diverse photothermal‐active ultrathin membranes, which included Au nanorods along with a poly (N‐isopropylacrylamide‐co‐acrylamide) copolymer on SWCNTs have been developed by Liang Hu et al.^[^ ^63^ ^]^ Such nanoporous membranes are characterized by their ability to efficiently separate oil from water nanoemulsions. High flux was achieved with values reaching 35 890 m^3^ m^−^^2^·h·bar owing to them underwater oleophobic and hydrophilic surface characteristics in combination with nanometer scale pores. In addition, the copolymer and Au nanorods also allow for light control of the membrane flux due to their thermally responsive materials. Membranes also exhibit very high separation efficiency, greater than 99.99% along with anti‐fouling and anti‐friction properties, and reusability capabilities. The effectiveness of dithiocarbamate anchor polymer/Oregano smectite composites as sorbents for removing mercury from aqueous liquids was studied by Say et al.^[^ ^64^ ^]^ By examining their FT‐ IR spectra, the synthesized nanocomposites were identified. After that, carbon disulfide was used to react with the modified smectite nanocomposites and give the organoclay nanosheet dithiocarbamate functional groups. Hg (II), C_6_H_5_Hg(I), and CH_3_Hg(I) were removed using these dithiocarbamate‐anchored compounds. The adsorption limits for CH_3_Hg(I) and Hg (II) were 157.3 mg g^−1^ and for C_6_H_5_Hg (I) were 90.3 mg g^−1^. Based on solutions containing all three mercury ions, the adsorption capabilities were 12.7 mg g^−1^ for C_6_H_5_Hg(I), 9.2 mg g^−1^ for CH_3_Hg(I), and 7.7 mg g^−1^ for Hg (II). A thin‐film nano composite (TFN) membrane has been engineered to improve the adsorption of low‐molecular‐weight organic micropollutants (OMPs) in water via Nadeem Baig et al.^[^ ^65^ ^]^ Enhancement in cross‐linking capability of the GO was achieved through amino silane functionalisation. The core analysis showed that the GO is 2–3 layers thick, with the amino silane functionalized GO appearing to be multiple thin layers. Functionalized GO was incorporated into TFN membranes, resulting in enhanced hydrophilicity in comparison to traditional TFC membranes, and SEM and FTIR were employed to verify the functionalized GO incorporation into TFN membranes. Furthermore, the removal efficiencies of OMPs water pollutants achieved by the commercially available TFN membrane substrates with functionalized GO have been positively tested: roughly 100% of removed Bisphenol‐A, 90% of Caffeine and ATT HCl, and ≈80% for acetaminophen. The TFN membrane approach presented herein has a good prospect of use for nanoporous membranes in water purification processes targeting resistant organic pollutant substances. Chitosan/rectorite compounds were studied by Feng et al.^[^ ^66^ ^]^ for their capacity to adsorb. They chose Acid Red (AR) dye as a model of pollutant. FTIR and XRD were used to describe the prepared nanocomposite. The maximum AR taken using chitosan/rectorite composites is 95.82 mg g^−1^. Fisli et al.^[^ ^67^ ^]^ developed magnetite‐silica (Fe_3_O_4_/SiO_2_) nanocomposites as adsorbents to successfully MB should be eliminated from the water specimen. These materials were developed, described, and assessed. Silica‐encapsulated magnetite was produced using the sol–gel method, and Fe_3_O_4_ was produced by co‐precipitating ferrous and ferric chloride ions in the presence of NH_4_OH. This synthesized material was characterized using different tools, including the TEM, Zeta potential meter, VSM, and XRD. The characterization's findings demonstrated that coating the prepared Fe_3_O_4_ nanoparticles with silica to create Fe_3_O_4_/SiO_2_ nanocomposite was effective. To examine the effectiveness of their adsorption, magnetic silica nanoparticles' capacity to absorb methylene blue dye in an aquatic solution was used. When using Fe_3_O_4_/SiO_2_ with a 3:1 ratio of iron to silica, shaking for 5 h, the residual MB in solution was 13.3%, 30.2% in ratio (2:1), and 24.2% for an initial concentration of 20 mg L^−1^ MB (1:1). Utilizing an external magnetic rod, easily recovered from treated water, the composite effectively adsorbs methylene blue in water. Chitosan@iron@silver (CS@Fe@Ag) nanocomposites were created by Olajire et al.^[^ ^68^ ^]^ developed a simple green synthesis technique. Chitosan@iron@silver (CS@Fe@Ag) can be used as a natural coagulant in Effluent Treatment Plants (ETPs) that treat wastewater with the least chance of organic release, making it the best alternative to the synthetic polyelectrolytes that have traditionally been used in ETPs. Equimolar amounts of the Fe@Ag core shell bimetallic nanoparticles (NPs) were created at 100 °C by adding Alchornealaxiflora leaf extract to their salt mixture in equimolar doses. (SEM), (FTIR), (XRD), and UV‐ Vis methods were used to characterize the resulting bimetallic nanocomposite and nanoparticles. The binding of the bimetallic shell (Ag shell) with the R‐COOH to produce AgO was confirmed by the FTIR spectra, XRD, and, UV–vis, providing evidence for the creation of Fe @Ag inside the matrix of chitosan polymer. To determine water quality parameters, they examined the change in pH value, amount of catalyst and stirring duration on reducing the wastewater turbidity. Under optimal conditions, the prepared nanocomposite produced greater value COD (15.5%), TDS (54.9%), and BOD (48.7%) compared with pure CS, which gave COD (3.6%), TDS (38.9%), and BOD (25.5%). To increase the membrane's antifouling resilience to oil deposits, Moeinzadeh et al.^[^ ^69^ ^]^ synthesized ultrafiltration (UF) nanocomposite membranes along with various concentrations of nanocrystalline cellulose with amino functions (NCs). Results of the characterization showed that the nanocomposite membranes' Despite pore size reduction, the addition of NCs significantly improved overall porosity and hydrophilicity. TEM was used to analyse nanoparticles structure for before and after the modification of the surface. FT‐IR used for examining the membrane surface's functional groups. Surface and cross‐sectional pictures were captured using SEM. In accordance with the UF study findings, when 1 wt. % of NCs were added, the membrane of nanocomposite increased water flux at a rate that was 43% higher than the pure membrane. For one to four rounds, a 250‐ppm emulsion solution of oil‐in‐water is treated. Nanocomposite membrane exhibited excellent water recovery rates of 85%, 98%, and (>98.2%) as well. An encouraging level of oil resistance.

The efficiency of using activated carbon and chitosan nanocomposite to clean up phosphate, ammonia, and nitrite pollutants from fishponds in Aq‐Qala was studied by Rezaei et al.^[^ ^70^ ^]^ Utilizing emission scanning electron microscopy and FTIR. The results showed that under ideal circumstances, including pH 7, 50 mg L^−1^ of wastewater is present, and the contact time is 60 min. The phosphate, ammonia, and nitrite contaminants had the most significant removal efficiency and adsorption capacities of 99.77%, 65.65% and 99.98%, 6.14, 7.32, and 6.65 mg g^−1^, respectively. Because the chitosan and activated carbon nanocomposite had a high clearance rate (99.98%), the adsorbent was very effective at removing pollutants. (nitrite, phosphate, and ammonia). By using a polymer grafting technique, Shawky et al.^[^ ^71^ ^]^ created multiwall element nanotubes (MWCNT)/aromatic polyamide (PA) nanocomposite membranes. The resulting nanocomposite membranes' surface morphology, coarseness, and mechanical substance were characterized by (SEM), (AFM), and micro stress analysis, respectively. As evidenced by AFM and SEM measurements, MWCNTs were uniformly spread throughout the PA matrix. Comparing the MWCNT addition to the basic case, 10% PA membrane, salt, and organic material rejection were also improved. According to SEM images, even at the highest concentration, the MWCNTs which developed by functionalization process in the produced film were evenly dispersed and well mixed throughout the polymer matrix, the CNTs were dispersed irregularly in past attempts to make CNT/polymer composite membranes. Humic acid removal through the MWCNT composite membranes rose from 54% to 90% as MWCNT loading increased from 0 to 10 mg g^−1^. Wang et al.^[^ ^72^ ^]^ created a magnetic nanocomposite based on graphene employed as an adsorbent to remove pigment fuchsin from aqueous solutions. This magnetic graphene hybrid (G/Fe_3_O_4_) was created by applying the chemical in situ co‐precipitation method. The prepared materials were fully characterized using XRD and SEM. To examine the adsorption properties of this material, they used fuchsine, an organic pigment, as a model. As a result, the dye's clearance rate reaches 99.4% from 82.2%. Gong et al.^[^ ^73^ ^]^ created a magnetic multi‐wall carbon nanotube (MMWCNT) nanocomposite as an adsorbent to remove the cationic pigments from the water solution. The characteristics of the MMWCNT nanocomposite material were characterized using XRD, SEM, and BET. The synthesized material adsorption properties were evaluated using BCB, NR, and MB as adsorbates. In order to remove cationic dyes from effluent using the MMWCNT nanocomposite adsorbent, the amount of catalyst was raised from 0.3 to 0.9 g L^−1^. As the amount of catalyst was raised, it was seen that the percentages of dyes adsorbed rose. The removal ratio of dyes increased in NR to 98.33% from 17.11%, BCB to 98.8% from 17.6%, and MB to 99.16% from 30.1%. The findings revealed that the absorption of NR was 20.33 mg g^−1^, BCB was 23.55 mg g^−1^, and MB15.74 mg g^−1^ by using the prepared material. Yang et al.^[^ ^74^ ^]^ prepared magnetic Fe_3_O_4_‐activated carbon nanocomposite samples and used them to eliminate methylene blue from water. TEM was used to analyze them, and powder XRD was used to gauge the samples. The pore structures of Rice Husk based activated carbon (RHC) and RHC‐Fe_3_O_4_ were investigated using N_2_ adsorption/desorption analysis. magnetic properties were examined by VSM technique. They investigated if RHC‐Fe_3_O_4_ could remove MB from water solution. The created materials have excellent magnetic separation efficiency, exhibiting increased adsorption capacity and strong affinity even with 23 weight percent Fe_3_O_4_ within the magnetic activated carbon. It is anticipated that the magnetic RHC‐Fe_3_O_4_ that was created will show promise as a sorbent to remove various toxic pollutants from wastewater.

Using an in situ polymerization process, Rachns et al.^[^ ^75^ ^]^ produce a ZnFe_2_O_4_‐PANI nanocomposite and then use XRD and SEM to characterize it. The electrical conductivity and the dielectric constant have been measured to describe it at various temperatures and frequencies. The nanocomposite successfully eliminated the RHB dye. The findings showed that at a dye concentration of 2 ppm, there was a maximum removal (99%) after 40 min. The highest amount of dye was eliminated at pH 2, and the least at pH 10. The removal was reduced by adding NaCl to the aqueous solution. The adsorption process was exothermic and spontaneous, according to thermodynamic metrics.

By burning Gundelia Tournefortii straw, Yang et al.^[^ ^76^ ^]^ created magnetic‐activated carbon composites easily and affordably. Several factors were used to assess how the magnetic‐activated carbon nanocomposite affected the Cr (VI) adsorption from water. Studies on the adsorbent's SEM, VSD, UV, FTIR, and XRD properties have been conducted. The generated nanocomposite was ferromagnetic, according to the results. They demonstrated the potential of magnetic activated carbon nanocomposite as an absorbent to eliminate chromium ions from water. The following results were achieved as the ideal removal conditions: The concentration was equal to 50 ppm, pH 5, 0.03 g of adsorbent amount, and the temperature was 25 °C. The results showed that more than 90% of Cr (VI) was removed. Rapid metal removal is very important practically because it allows for the efficient and economical use of tiny adsorbent volumes. Cr (VI) is successfully removed from the aqueous solution by artichoke straw particles using the cleaning solution of synthetic sewage.

A Halloysite composite was used by Purnima et al.^[^ ^77^ ^]^ to efficiently remove manganese from water through adsorption. The researchers optimized experimental parameters, including dosage, contact time, pH levels, and initial Mn concentration, to create an environmentally friendly material for practical manganese removal. The composite's crystallinity increased from 70.88% to 77.4% after adsorption, indicating successful adsorption. At a transition temperature of 303 K, the composite showed an impressive 93% manganese removal efficiency. Comprehensive analyses were conducted using various instruments to compare the composite's performance before and after adsorption. Functionalized GO nanosheets introduced additional water pathways in the active layer, increasing water permeate flux by ≈25% compared to pristine TFC and non‐functionalized GO TFN membranes. The TFN membrane with functionalized GO showed superior removal efficiencies of organic pollutants found in water, achieving nearly 100% removal of Bisphenol‐A (BPA), ≈90% for Caffeine (CFN), Amitriptylene HCl (ATT HCl), and ≈80% for Acetaminophen (ACT). This TFN membrane design offers significant potential for water purification applications, particularly in removing challenging organic contaminants.

**Figure 5:**
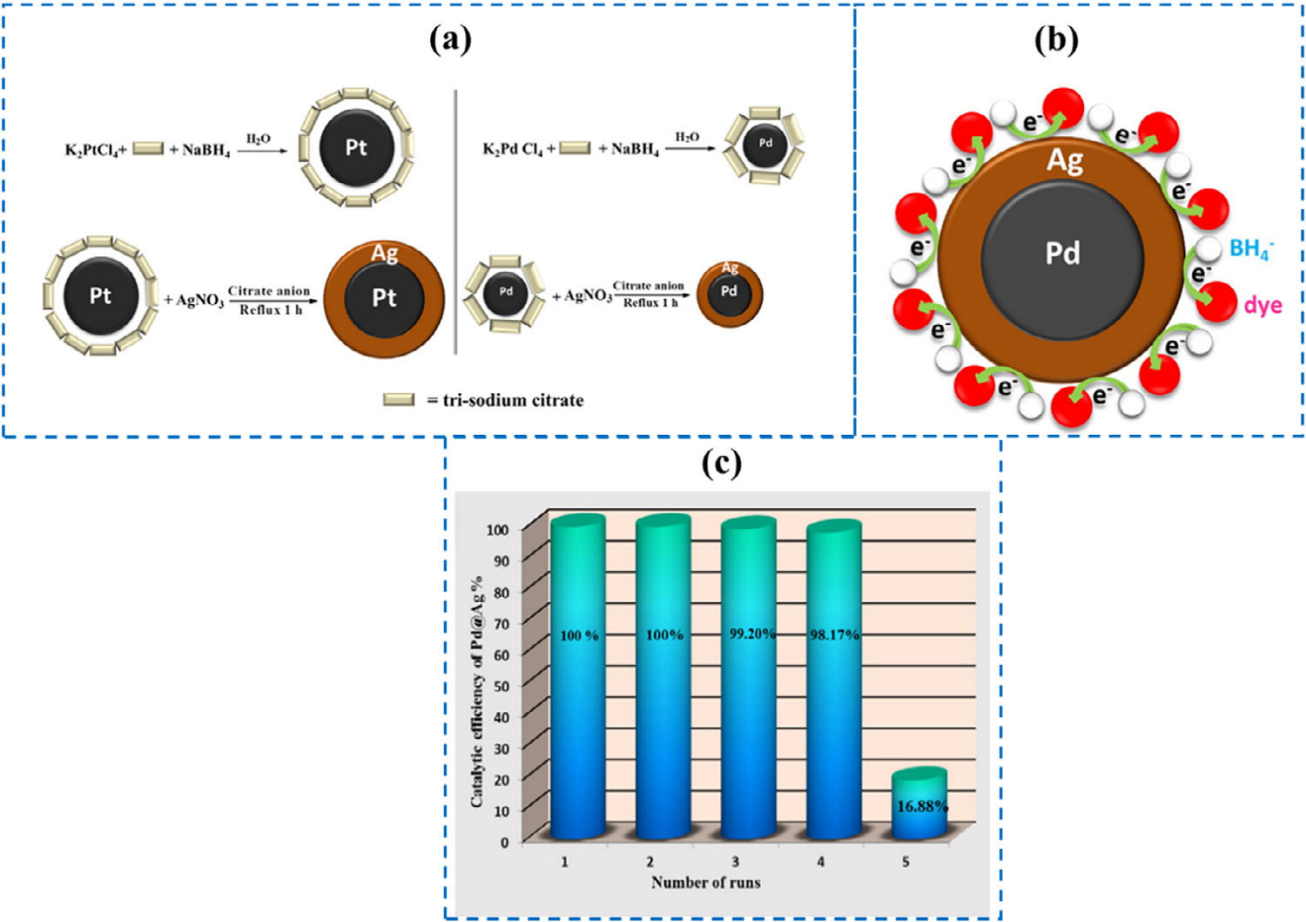
a) Synthesized Pt@Ag and Pd@Ag core/shell nanoparticles, b) The core/shell structure acts as an electron transfer mediator between NaBH4 and dye molecules, c) The catalytic performance of Pd@Ag was evaluated over five consecutive cycles of dye reduction. With permission from ref. [79] Copyright (2018) Elsevier.

Bakr et al.^[^ ^78^ ^]^ Synthesized platinum/silver (Pt@Ag) and palladium/silver (Pd@Ag) core/shell nanoparticles through a two‐step citrate reduction method, as shown in **Figure** **5**a, tracking their formation using UV–vis spectroscopy. Transmission electron microscopy showed that these nanostructures are spherical, with average sizes of ≈32.17 nm for Pt@Ag and 8.8 nm for Pd@Ag. Further characterization by FT‐IR and XRD confirmed their structure and composition. To evaluate their catalytic capabilities, the researchers tested these nanoparticles in the reductive degradation of Congo red dye, where the core/shell particles acted as electron mediators, facilitating electron transfer from sodium borohydride (NaBH_4_) to the dye molecules (as see in Figure 5b).  They systematically studied how factors like catalyst dosage, concentrations of the dye, and NaBH_4_ affected the degradation efficiency. Comparative analysis revealed that Pd@Ag exhibited superior catalytic performance compared to Pt@Ag, which was attributed to its smaller particle size and larger surface area, providing more active sites. Furthermore, the catalytic performance of Pd@Ag was evaluated over five consecutive cycles of dye reduction, demonstrating excellent stability and reusability as shown in Figure 5c. These findings highlight Pd@Ag core/shell nanoparticles as promising, cost‐effective catalysts for environmental applications, particularly in removing dye pollutants from wastewater.

**Figure 6:**
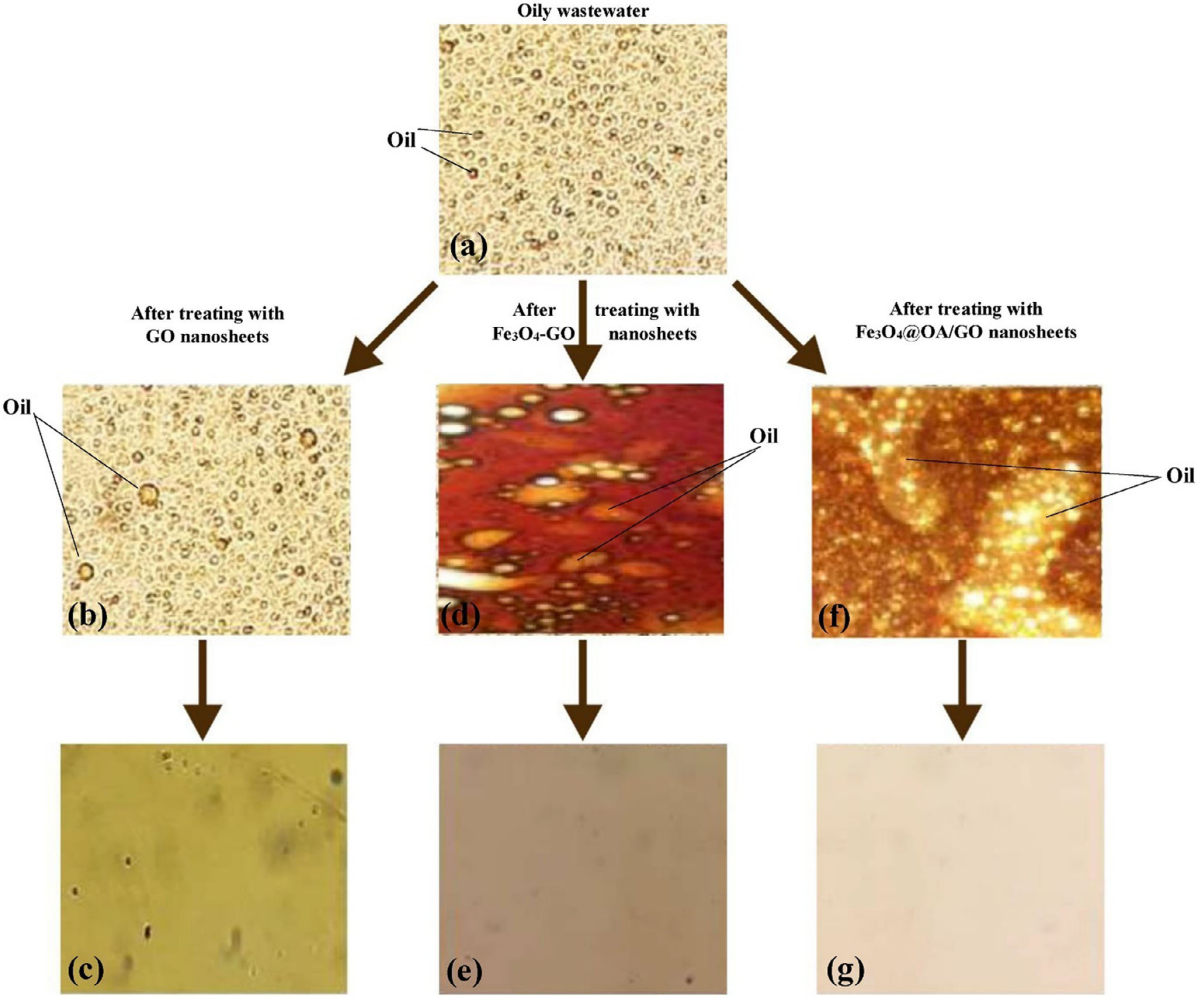
Images captured using optical microscopy show the oil droplets in the emulsion before (a), after demulsification, (b) GO, and (d) Fe3O4‐GO, (f) Fe3O4@OA/GO nano emulsifiers, and separated waters (c), (e), and (g) after demulsification with each of the nano emulsifiers at 25 °C. With permission from ref. [80] Copyright (2019) Elsevier.

In order to handle oily wastewater, Javadianet et al.^[^ ^80^ ^]^ Synthesized graphene oxide (GO)‐based nanosheets that are eco‐friendly, inexpensive, highly effective, and recyclable. SEM, XRD, CA, FT‐IR, TEM, and VSM were employed to examine the shape, physical and chemical characteristics of GO, Fe_3_O_4_‐GO, and Fe_3_O_4_@ oleic acid (OA)/GO nanosheets. According to the study, the Fe_3_O_4_@OA/GO hydrophobic nano emulsifiers perform admirably, demulsifying with a 99.99% effectiveness rate in just a few seconds. The effectiveness of the nano emulsifiers Fe_3_O_4_‐GO and Fe_3_O_4_@OA/GO could also be reused up to six times without significantly losing their effectiveness. Additionally, the impact of different temperatures on the ability of nano‐emulsifiers based on graphene oxide to demulsify was investigated. The implicit temperature findings significantly affected the instability of oily wastewater because temperature increases made the emulsified oily wastewater unstable. As shown in **Figure** **6**, the amount of oil in the separated water was significantly reduced because of the separation of oil flakes from oily wastewater using GO‐based nano emulsifiers.

Nodeh et al.^[^ ^81^ ^]^ effectively created and used the magnetic Fe_3_O_4_@SiO_2_/GO as an adsorbent to simultaneously remove as (III) and as (V) from a variety of natural sources of water. The synthesized adsorbent was described using FESEM, FT‐IR, and XRD. The Fe_3_O_4_@SiO_2_/GO showed strong (III and V) adsorption abilities at pH equal 4. In the pH range from 7 to 10, the adsorbent only demonstrated selective (III) absorption. The adsorption capacity for As III was 7.51 mg g^−1^, and as(V) was 11.46 mg g^−1^ due to the high adsorption effectiveness (at 0.05 ng mL^−1^ for as (III) and as (V) concentration reached > 95%). With the prepared material, (III) has a low LOD (28.0 pg mL^−1^). This synthetic nanocomposite is an excellent substitute material for the removal of as (III) and as (V) from samples collected from water. The material has a built‐in ability to repeat and be selective. This adsorbent has quick extraction (or adsorption), excellent regenerated (12 times), and is economically advantageous.

Nay et al.^[^ ^82^ ^]^ developed an effective absorbent (P@SiO_2_) through a hydrothermal process. The dye MB was chosen for kinetic tests on the P@SiO_2_ nanocomposite's removal behavior. The morphologies of the nanocomposite were confirmed by SEM. XRD was used to explain the crystal structure of the composite. FT‐IR of the prepared nanoparticles was examined. The experiment's findings demonstrated quick kinetic adsorption, reaching equilibrium in less than 100 s. From the results, a gram of P@SiO_2_nanocomposite can also get rid of 76.92 mg of the dye MB. The process of adsorption was exothermic, spontaneous, and ordered at the solid/solution interface, according to thermodynamic studies. Finally, the adsorption activity of MB dye did not change by sodium chloride's presence.

Salem et al.^[^ ^79^ ^]^ Platinum/silver (Pt@Ag) and palladium/silver (Pd@Ag) core/shell nanoparticles were created using the citrate method. They evaluated the synthesized elements using TEM, FT‐IR, and XRD. They examined the catalytic activity of the created core/shells in the catalytic reduction of CR dye. From the results, they found that by raising the concentration of NaBH_4_ and the amount of catalyst, the reaction rate was enhanced. When the CR dye concentration was varied, the rate of reaction increased at low concentrations of CR dye and then decreased at higher concentrations of CR dye. Additionally, it was found that the percentage of degradation after 6 min was 85% when Pd@Ag was used as the catalyst and 30% when Pt@Ag was used as the catalyst. By making a comparison between the two prepared materials, they found that Pd@Ag had a higher catalytic efficiency than Pt@Ag.

Superparamagnetic AgFeO_2_@polypyrrole/SiO_2_ was synthesized by El‐Attar et al.^[^ ^83^ ^]^ using a three‐step process. XRD, SEM, VSM, EDX, TEM, TGA, and FTIR were chosen to characterize the synthesized samples. In comparison to the prepared materials, they found that AgFeO_2_ has 0.1365 min^−1^, AgFeO_2_@PPy has 0.2274 min^−1^, and AgFeO_2_@PPy/SiO_2_ has 0.9209 min^−1^values. These results proved that the nanocomposite has the highest rate constant value for chromatrope 2R reduction. Significant catalytic activity was shown for AgFeO_2_@PPy/SiO_2_ toward catalytic (oxidation or reduction) to vanish some dyes in aqueous solution, including chromotrope 2R, tartrazine, AB, MV 2B, and MB. The percentages of degradation for dyes in catalytic reduction were 98.64% for tartrazine, 98.17% for MB, and 99.38% for chromotrope 2R, and for catalytic oxidation were 99.34% for AB, 91.79% for MV 2B, and 75.84% for MB. The AgFeO_2_@PPy/SiO_2_ nanocomposite's recovery and reusability demonstrated high stability and constant catalyst effectiveness for thirteen cycles during catalytic reduction processes and nine cycles during catalytic oxidation processes. The research results have shown that the synthesized materials have a strong probability of removing all the tested pigments from an aqueous solution.

**Figure 7:**
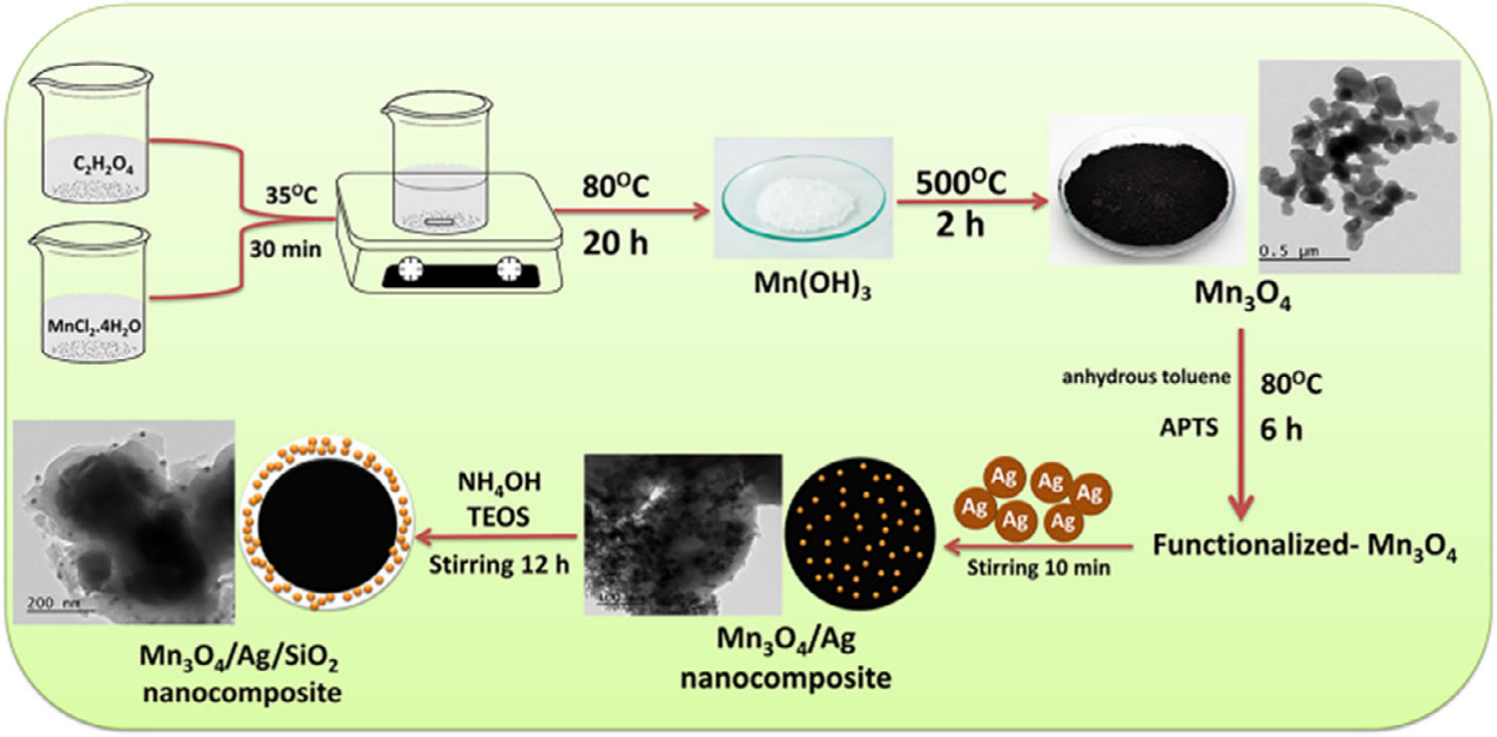
Preparation of Mn3O4/Ag/SiO2 nanocomposite. With permission from ref. [84] Copyright (2020) Elsevier.

An effective heterostructurenano catalyst made of a Mn_3_O_4_ core and a SiO_2_ shell impregnated with silver nanoparticles was described by Bakr et al.^[^ ^84^ ^]^ as given in **Figure** **7**. The triple nano catalyst Mn_3_O_4_/Ag/SiO_2_ was synthesized in three easy steps. FT‐IR and XRD were used to examine the structure. TEM and SEM were used to determine the surface morphology. They investigated how these materials affected DB 78′s degradation. Adding an extra dye, the synthesized nanocomposite's catalytic activity was examined for a binary system (DB 78 and SY), which was completely degraded, demonstrating excellent catalytic activity for the triple nanocomposite. These pigments had degradation rates of 99.33 and 94.68%, respectively. In the DB 78 reduction process, they explored how well the triple nanocomposite recovered and could be reused. They found that there were five recovery reactions done. Over four cycles, it demonstrated excellent stability and consistent catalyst effectiveness. All previous results in **Table** **1** cover the various synthesized nanocomposites used in wastewater treatment.

**Table 1:** Nanocomposites in wastewater treatment.

Authors	Nano catalysts	Ligand	Active sites	Synthesis methods	Characterization techniques	Contaminant	Degradation/adsorption/uptake rate efficiency
Ejaz Hussain et al.^[^ ^60^ ^]^	PANI/GO/MOF‐Fe_3_O_4_ nanocomposite	PANI	Carboxylic (‐COOH), hydroxyl (‐OH), and amine (‐NH_2_) groups	In situ oxidative polymerization	FTIR, XRD, SEM, TGA, BET, and XPS	Methyl Orange (MO) and Naproxen Sodium (NAP)	Adsorption capacities achieving are 239.78 mg g^−1^ for MO and 40.64 mg g^−1^ for NAP.
Anirudhan et al.^[^ ^61^ ^]^	Polyamidoxime/Organo‐bentonite (PAO/Organo‐B)	Amidoxime	‐C(NH_2_) = NOH	In situ intercalation polymerization technique	XRD, SEM, FT‐IR, surface area analyser, thermogravimetry, and potentiometric	Cu (II), Zn (II), and Cd (II)	A peak adsorption efficiency of 99.8% for Cu (II), 98.9% for Zn (II), and 97.4% for Cd(II) was achieved using 2 g L^−1^ of adsorbent at a pH of 6.0, with the initial metal concentration set at 25 mg L^−1^.
Anirudhan et al.^[^ ^62^ ^]^	Humic humic acid‐immobilized amine‐modified polyacrylamide/bentonite (HA‐Am‐PAA‐B)	Humic acid	Carboxylic and amine groups	Polymerization and then immobilizing humic acid onto the amine‐modified composite.	SEM, XRD, EDX, and FT‐IR	CV, MB, and MG	The highest dye consumption was observed for MG at 199.4 mol g^−1^ (99.7%), followed by MB at 193.4 mol g^−1^ (96.7%), and then CV at 187.5 mol g^−1^ (93.8%) when an initial dye concentration of 400 mol L^−1^ was applied.
Liang Hu et al.^[^ ^63^ ^]^	Au nanorods/poly(N‐isopropylacrylamide‐co‐acrylamide) cohybrid single‐walled carbon nanotube (SWCNT) nanoporous membranes	Polydopamine for anchoring SWCNTs and pNIPAm‐co‐AAm copolymers	Gold nanorods/ Polydopamine‐coated SWCNTs	Multi‐step hybrid synthesis process	Raman analysis, SEM, XPS, TEM, XRD, FTIR, UV–vis spectroscopy, and thermal analysis.	Oil	Ultrahigh separation efficiency (over 99.99%)
Say et al.^[^ ^64^ ^]^	Dithiocarbamateanchor polymer/organosmectite composites	Dithiocarbamate groups	Dithiocarbamate (‐CS_2_^−^)/ Amine (‐NH_2_)/ Benzyl group (‐C₆H₅CH_2_)/ Hydroxyl (‐OH)/ Quaternary ammonium groups (‐NR_4_⁺)	Polymerization/ multi‐step hybrid synthesis process	FT‐ IR spectra	Mercury in various forms	The adsorption capabilities for CH_3_Hg(I) = 214.6 mg g^−1^, C_6_H_5_Hg(I) =, 90.3 mg g^−1^ and Hg (II) = 157.3 mg g^−1^.
Nadeem Baig et al.^[^ ^65^ ^]^	Thin‐film nanocomposite (TFN) membrane containing functionalized graphene oxide nano sheets	GO	(Hydroxyl ‐OH, carboxylic ‐COOH, and amine ‐NH)	Functionalization of graphene oxide and then interfacial polymerization	TEM, SEM, FT‐ IR, XPS, and contact angle measurements	low‐molecular‐weight OMPs	≈100% removal for Bisphenol‐A (BPA), ≈90% for Caffeine (CFN) and Amitriptyline HCl (ATT HCl), and ≈80% for Acetaminophen (ACT).
Feng et al.^[^ ^66^ ^]^	Chitosan/rectorite composites	Chitosan	Amino (‐NH_2_) and hydroxyl (‐OH) groups	Multi‐step hybrid synthesis process	FTIR and XRD	Acid red (AR)	95.82 mg g^−1^
Fisli et al.^[^ ^67^ ^]^	Magnetite‐silica (Fe_3_O_4_/SiO_2_) nanocomposites	Silica	Silanol groups (‐Si‐OH)‐ Iron oxide (Fe_3_O_4_)	Combination of co‐precipitation and sol–gel coating	TEM, Zeta potential meter, VSM, and XRD	Methylene Blue (MB)	The residual MB in solution was 13.3% for a ratio of 3:1, 30.2% for 2:1 and 24.2% for 1:1 iron to silica ratio in Fe_3_O_4_/SiO_2_.
Olajire et al.^[^ ^68^ ^]^	Chitosan@iron@silver (CS@Fe@Ag) nanocomposites	Chitosan	Iron, Silver, and Amino, Hydroxyl, and Carboxyl groups	Facile green synthesis method	SEM, FTIR, XRD, and UV‐ Vis	BOD, COD, and TDS	COD (15.5%), TDS (54.9%) and BOD (48.7%). under ideal circumstances.
Moeinzadeh et al.^[^ ^69^ ^]^	Ultrafiltration (UF) nanocomposite membranes along with various concentrations of nanocrystalline cellulose with amino functions (NCs)	Nanocrystalline Cellulose	Amino functional groups/ Hydroxyl groups	Phase inversion method combined with nanocrystal functionalization and incorporation	SEM, FTIR, and TEM	Oil	This nanocomposite membrane demonstrated strong oil rejection capabilities, exceeding >98.2%, along with impressive water flux recovery rates of ≈98% after the first cycle and ≈85% after four cycles when processing a 250 ppm oil‐in‐water emulsion solution.
Rezaei et al.^[^ ^70^ ^]^	Chitosan and activated carbon nanocomposite	Chitosan and AC	Amino groups (‐NH_2_)/ Hydroxyl groups (‐OH)	Chemical activation and composite formation process, which combines pyrolysis‐based carbon activation with polymer composite fabrication	FTIR and SEM	PO_4_, NO_3_, and AH_3_	Chitosan and activated carbon nanocomposite had a high clearance rate (99.98%), the adsorbent was very effective at removing pollutants (nitrite, phosphate, and ammonia).
Shawky et al.^[^ ^71^ ^]^	Multiwall element nanotubes (MWCNT)/aromatic polyamide (PA) nanocomposite	MWCNTs	Polar amide groups and aromatic sites	Polymer grafting method	SEM, AFM, and micro stress analysis	Humic acid, salt	Humic acid removal by the MWCNT composite membranes improved from 54% to 90% as the MWCNT content rose from 0 to 10 mg g^−1^. The salt rejection rate increased significantly from 24% to 64.5%, while both permeability and specific flux declined, dropping from 0.76 to 0.28 L m^−^^2^·h·bar and from 32 to 11 L m^−^^2^·h, respectively.
Wang^[^ ^72^ ^]^	Graphene‐based magnetic nanocomposite G/Fe_3_O_4_	Graphene	Fe_3_O_4_ nanoparticles	In situ chemical co‐precipitation	XRD and SEM	Organic dye	From 82.2 to 99.4%, the pigment was removed. Over 0.4 g L^−1^ of G/Fe_3_O_4_.
Gong et al.^[^ ^73^ ^]^	A nanocomposite made of magnetic multi‐wall carbon nanotubes	1‐(2‐Pyridylazo)‐2‐naphthol (PAN)	Magnetic nanoparticles/ Carboxylic (‐COOH) and hydroxyl (‐OH) groups	In situ chemical co‐precipitation combined with surface functionalization.	XRD, SEM, and BET	MB, NR, and BCB	1.0 µg L^−1^ for Pb(II) and 0.6 µg L^−1^ for Mn(II).
Yang et al.^[^ ^74^ ^]^	Magnetic Fe_3_O_4_ @activated carbon nanocomposite	Rice husk‐derived	Fe_3_O_4_ nano particles/Activated Carbon Pores	Combination of chemical activation and in situ thermal reduction	TEM, powder XRD, N_2_ adsorption/desorption analysis, and VSM technique	MB	A significant adsorption capacity of 321 mg g^−1^ for Methylene Blue (MB) from water solution.
Rachns et al.^[^ ^75^ ^]^	ZnFe_2_O_4_‐PANI nanocomposite	PANI	Zinc Ferrite (ZnFe_2_O_4_)/ Polyaniline functional groups	In situ polymerization method	XRD and SEM	Rhodamine B dye (RHB)	ZnFe_2_O_4_ alone: 59% dye removal. ZF‐PANI nanocomposite: 85% dye removal.
Yang et al.^[^ ^76^ ^]^	Magnetic activated carbon nanocomposite	AC	Iron Oxide Nanoparticles/ Activated Carbon Pores	Facile green synthesis method	SEM, VSD, UV, FTIR, and XRD	Chromium (VI)	After 120 min is enough to remove more than 90% of Cr (VI) at an optimal dosage of 0.03 g, pH 2.03, and 50 ppm Cr(VI) concentration.
Purnima et al.^[^ ^77^ ^]^	Halloysite‐cerium (HNT‐Ce) nanocomposite	halloysite	Cerium Oxide (CeO_2_), and Hydroxyl (‐OH) groups	Hydrothermal synthesis combined with chemical functionalization	SEM, EDS, TEM, FT‐IR, XRD, and UV–vis	Mn^2+^	At a transition temperature of 303 K, the composite achieved a remarkable 93% efficiency in removing manganese, showcasing its high effectiveness under these particular conditions.
Bakr et al.^[^ ^78^ ^]^	Colloidalcore‐shell nanoparticles of Ag@Pd (NPs)	Trisodium citrate	Palladium (Pd) Shell, and Silver (Ag) Core	Successive Reduction Method	TEM, UV, FTIR, and XRD	Direct Blue 14, CR, and Sunset yellow	Direct Blue 14 had a degradation effectiveness of 94.57%, which was higher than Congo Red's (90.23%), then Sunset Yellow's (83.56%).
Javadian et al.^[^ ^80^ ^]^	Fe_3_O_4_@OA/GO	Oleic Acid (OA)/ Graphene Oxide (GO)	Carboxylic (‐COOH) and hydroxyl (‐OH) groups/ Fe_3_O_4_ nanoparticles	Modified Hummers method	SEM, XRD, CA, FT‐IR, TEM, and VSM	Oil	Within a few seconds, 99.99% of the oil is removed.
Nodeh et al.^[^ ^81^ ^]^	Magnetic Fe_3_O_4_@SiO_2_/GO	Silica (SiO_2_)/ Graphene oxide (GO)	Fe_3_O_4_ magnetic nanoparticles/ Carboxylic groups (‐COOH)/ Hydroxyl groups (‐OH)/ Epoxy groups (‐C‐O‐C)	Modified Hummers method	FESEM, FT‐IR, and XRD	As (III) and As (V)	Demonstrated substantial adsorption capacities of 7.51 mg g^−1^ for As (III) and 11.46 mg g^−1^ for As(V) at a pH of 4.0.
Nay et al.^[^ ^82^ ^]^	Phosphate‐embedded silica (P@SiO_2_) nanocomposite	Phosphate Ions (PO_4_^3^^−^)	Silanol (‐Si‐OH) groups/ Phosphate (PO_4_^3^^−^) groups	Hydrothermal Route	SEM, XRD, and FT‐IR	Methylene Blue (MB)	Removal of 76.92 mg of the MB dye.
Salem et al.^[^ ^79^ ^]^	Pd‐Ag and Pt‐Ag (core@shell)	Trisodium citrate	Palladium (Pd), silver (Ag), and platinum (Pt)	Two‐step successive reduction method:	TEM, FT‐IR, and XRD	Congo Red (CR)	The percentage of degradation for CR dye after 6 min 85% when Pd@Ag used as catalyst and 30% when Pt@Ag used as catalyst. Pd@Ag had a higher catalytic efficiency than Pt@Ag.
El‐Attar et al.^[^ ^83^ ^]^	AgFeO_2_@Polypyrrole/SiO_2_ nanocomposite	Polypyrrole (PPy)/ Silica (SiO_2_)	AgFeO_2_ core/ Polypyrrole shell(nitrogen sites)/ (‐OH) groups	Multi‐step process combining hydrothermal synthesis, oxidative polymerization, and sol–gel coating	XRD, SEM, VSM, EDX, TEM, TGA, and FTIR	Chromotrope 2R, Tartrazine, Methylene Blue, Methylene Blue, Aniline Blue, And Methyl Violet 2B	The percentages of degradation for dyes in catalytic reduction were 98.64% for tartrazine, 98.17% for MB, and 99.38% for chromotrope 2R and for catalytic oxidation were 99.34% for AB, 91.79% for MV 2B and 75.84% for MB.
Bakr et al.^[^ ^84^ ^]^	Mn_3_O_4_/Ag/SiO_2_ Nanocomposite	3‐aminopropyltrimethoxysilane (APTS)/ Silica (SiO_2_)	Mn_3_O_4_, Ag, and SiO_2_	Hydrothermal Synthesis Combined with Surface Functionalization and Sol–Gel Coating	FT‐IR, XRD, TEM, EDX, and SEM	Direct Blue 78 (DB 78) and Solvent Yellow (SY)	The synthesized nanocomposite's catalytic activity for a binary system (both DB 78 and SY) removal was 99.33% and 94.68%, respectively.

## 4 Photocatalytic Technique for Wastewater Treatment using Nanocomposite

**Figure 8:**
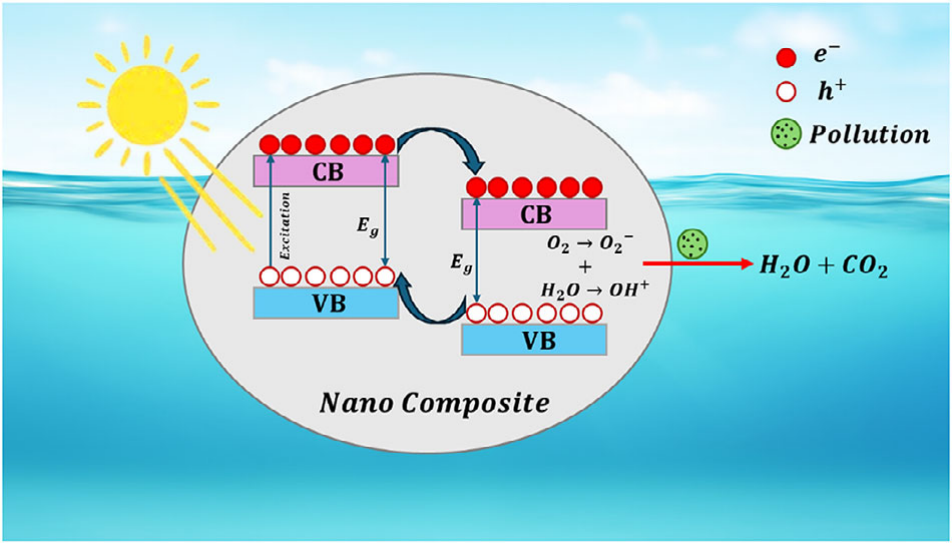
Mechanism of photocatalysis in water treatment using nanocomposites.

Photocatalysis using nanocomposites in water treatment is an advanced process that activates photocatalysts like TiO_2_ or ZnO with UV or visible light.^[^ ^85^, ^86^ ^]^ The core mechanism, as shown in **Figure** **8**. begins when the photocatalyst absorbs photons with energy (*h*ν) equal to or greater than its bandgap energy (*E_g_*), exciting electrons (*e* ^−^) from the valence band (*VB*) to the conduction band (*CB*), simultaneously generating holes (*h* ^+^) in the VB^[^ ^87^ ^]^ according to the Equation (1):

(1) $$\mathrm{Photocatalyst}+\mathrm{Light}\;\mathrm{Energy}\;\left(hv\right)\to e_{CB}^{-}+h_{VB}^{+}$$

These photogenerated charge carriers then migrate to the catalyst surface. At the surface, the holes react with water (*H*_2_ *O*) or hydroxide ions (OH^−^) to form highly reactive hydroxyl radicals (OH^•^), while the electrons react with adsorbed molecular oxygen (O_2_) to produce superoxide radical anions $\left(O_{2}^{•-}\right)$ according to the Equations ((2), (3), (4)):

(2) $$h_{VB}^{+}+H_{2}O\;\to \;OH^{•}+H^{+}$$

(3) $$h_{VB}^{+}+\;OH^{-}\to \;OH^{•}$$

(4) $$e_{CB}^{-}+O_{2}\to {\;O}_{2}^{•-}$$

These powerful reactive oxygen species $\left(OH^{•},O_{2}^{•-}\right)$ non‐selectively attack organic and inorganic pollutants (*P*), breaking them down into simpler, less harmful compounds, ultimately leading to their complete mineralization into carbon dioxide (CO_2_), water (H_2_O), and mineral acids as shown in Equation (5):

(5) $$\begin{array}{ccc} OH^{•}/O_{2}^{•-} & + & \;\mathrm{Pollutants}\;\left(P\right)\to \;\mathrm{Intermediate}\;\mathrm{Products}\to CO_{2}\\ & & +\;H_{2}O+\mathrm{Inorganic}\;\mathrm{Ions} \end{array}$$

Photocatalytic water treatment using nanocomposites harnesses light energy to break down harmful contaminants in water.^[^ ^88^, ^89^ ^]^ One of the key factors in this process is charge separation, which increases the lifespan of the photogenerated electrons and holes, enabling them to effectively degrade pollutants.^[^ ^90^ ^]^ Nanocomposites, with their large surface areas and diverse electronic structures, help prevent the recombination of these charge carriers, improving their efficiency. Additionally, bandgap engineering adjusts the energy difference between the valence and conduction bands, allowing photocatalysts to absorb a broader range of light, particularly visible light, thereby making better use of natural sunlight for pollutant degradation.^[^ ^91^ ^]^

Additionally, the use of heterojunctions, where two materials with complementary band structures are combined, facilitates better charge transfer while minimizing recombination. This design improvement is especially useful for degrading pollutants like dyes and heavy metals.^[^ ^92^ ^]^ Another important factor is the surface reactions. The photocatalytic activity occurs at the surface of the nanocomposite, where pollutants are adsorbed. By modifying the surface of the nanocomposite, by adding metal nanoparticles or functional groups, researchers can increase the number of active sites, which further boosts the degradation process.^[^ ^93^ ^]^

This is what researchers are working to improve. By enhancing charge separation, bandgap engineering, heterojunctions, and surface modifications, they aim to optimize the photocatalytic efficiency of nanocomposites for treating wastewater. As demonstrated in ongoing studies, these advancements are key to developing more effective solutions for removing pollutants like dyes, heavy metals, and pathogens.

The nanocomposite TiO_2_/ZnO/SiO_2_ (TZS) was synthesized using a sol–gel‐hydrothermal method at 180 °C for 24 h using water and a mixture of water‐ionic liquids (ILs). XRD analysis revealed anatase, zincite, and amorphous silica after calcination treatment at 450 °C. TZS synthesized using water‐ILs media had a greater surface area and narrower bandgap energy, making it practical for use in the visible light region. The anatase phase of TiO_2_ was stable even when heated up to 1000 °C. SEM‐EDX and TEM results showed that TZS synthesized with water had a sphere‐like shape, while water‐[BMPyrr][BF_4_] media had nanocoral, nanorod, and nanocubic‐like shapes. TZS synthesized with water‐[BMPyrr][BF_4_] media can remove Pb (II) ions up to 99.98% better than water media. The specific particle morphology, larger surface area, and stable anatase phase make it a promising material for photocatalysis applications.^[^ ^94^ ^]^

Romolini et al.^[^ ^95^ ^]^ demonstrate the successful application of SiO_2_ NPs, NH_2_‐SiO_2_ NPs, and Ag‐SiO_2_ nanocomposite for the photocatalytic remediation of water. The samples were distinguished from TEM, FE‐SEM, EDX, and FT‐IR spectra. The efficient production of mesoporous silica nanoparticles (SiO_2_ NPs) containing ‐NH_2_ groups, a mesostructured resembling a regular channel and an average diameter of 120 nm (NH_2_‐SiO_2_ NP). Amino‐grafted silica nanoparticles have Ag NPs with a diameter of 10 nm attached to their surface (Ag‐SiO_2_). Due to silica surface defects, the three materials showed electronic transitions in the UV range. However, for Ag‐SiO_2_ NPs, the visible absorbance spectrum broadening is caused by the metal nanostructures' surface plasmon resonance. Utilizing the model chemical 9‐anthracenecarboxylic acid (9ACA) to study aromatic pollutants, additional studies were carried out to assess the materials' photodegradation effectiveness. SiO_2_ NPs did not significantly affect the degradation of 9ACA when exposed to 313 nm light. However, NH_2_‐SiO_2_NPs significantly affected the degradation of the anthracene derivative, causing up to 30% of it to be destroyed, and 66% when Ag‐SiO_2_ is employed as a catalyst. Interestingly, activation of the photocatalyst in the Ag‐SiO_2_ NPs by 405 nm light leads to 60% photodegradation. It was easy to determine the photocatalytic function of the various material components and to gain an understanding of the photocatalytic mechanism by using various materials and irradiation sources. With a 17% efficiency, reactive oxygen species production is photosensitive to silica and silica colloids covered with Ag. The photocatalytic activity of silica‐silver nanocomposites is beneficial for breaking down aromatic molecules.

Babyszko et al.^[^ ^96^ ^]^ prepared SiO_2_/TiO_2_ photocatalysts by sol–gel. Different SiO_2_ weights (between 11.2 and 17.2% wt.%) were employed in the produced materials. The photocatalysts were investigated using XRD, SEM, FT‐IR, and UV–vis. For the first time, it was demonstrated that fumed silica may be employed as a silica precursor. SiO_2_dramatically reduced crystallite growth during the heat treatment while enhancing the material's total pore volume and specific surface area. Energy in the bandgap also changed. The photoactivity of selected samples was evaluated using methylene blue's degradation. The results demonstrated that the SiO_2_ addition to the TiO_2_ framework enhanced the photocatalytic capabilities. It was shown that the titanium dioxide modification with SiO_2_ applied both before and after the calcination phase increased the photocatalytic activity. Compared to the original TiO_2_ (7.19%), all the generated photocatalysts demonstrated higher activity, which only eliminated methylene blue to a maximum of 75.81%.

Kumar et al.^[^ ^97^ ^]^ presented the physical mixing method used to create the ZnO/Polyaniline (PANI)/reduced graphene oxide (RGO) ternary nanocomposite and studied photocatalytic effectiveness in breaking down MO in the presence of sunshine. Three methods are used: FT‐IR, XRD, and UV–vis to examine the created samples. For ZnO, PANI, and RGO, the optical bandgap was calculated using the Tauc diagram. MO dye, whose breakdown rate was 99% in 50 min, was used to test the photocatalytic experiments. It has been found that PANI (wt.%) increases the photocatalytic efficiency. The PANI might produce more charge carriers, which would slow down the recombination process. A first‐order pseudo‐reaction is compatible with the kinetic studies. The nanocomposite works as a powerful photocatalyst to rid water of organic impurities. Aslam et al.^[^ ^98^ ^]^Three processes were required to synthesize the nanocomposite. i)The extraction of silicon dioxide (SiO_2_) from rice hulls ii) the sol–gel synthesis of tin oxide‐silica (SnO_2_‐SiO_2_), and iii) the hydrothermal synthesis of samarium (Sm)/SnO_2_‐SiO_2_The Sm/SnO_2_/SiO_2_ nanocomposite. EDX, TGA, TEM, XRD, FT‐IR, and XRD were used to characterize them. SnO_2_‐SiO_2_ and Sm/SnO_2_‐SiO_2_ nanocomposites had bandgaps of 5.1 and 4.8 eV, respectively. Particle size, specific surface area, dislocation density, and poor gap were observed to decrease with the doping Sm on SnO_2_‐SiO_2_ nanocomposite. Under solar irradiation, the produced material's photocatalytic activity was examined against MB dye. From the findings, the degradation percentage of dye using SnO_2_‐SiO_2_ nanocomposite was 71%, and 78% for using Sm/SnO_2_‐SiO_2_ nanocomposite.

Al‐Rawashdeh et al.^[^ ^99^ ^]^ reported accomplished to creation of a successful photocatalyst based on graphene oxide (GO)/ZnO nanocomposites with embedded metal nanoparticles. The nanocomposites were examined using FT‐IR, SEM, and XRD. Due to the addition of metal nanoparticles, which improved charge transfer and photoactivity, the nanocomposites had better photocatalytic activity than GO/ZnO nanocomposites. After 90 min of sunlight exposure to the dye MB, a nanocomposite containing 3.125% GO demonstrated a catalytic activity of 84% MB. On the activity of the photocatalyst, the impacts of implanted copper and silver nanoparticles were examined. In the GO/ZnO/Cu nanocomposite, the activity of MB degradation dropped by 50%, but it considerably increased in the GO/ZnO/Ag nanocomposite and reached 100% after 40 min of exposure to sunlight. The considerable surface area of the nanocomposite, which was raised by the addition of GO, making it a powerful and adjustable photocatalyst for the photodegradation of organic pigments in wastewater from industries.

**Figure 9:**
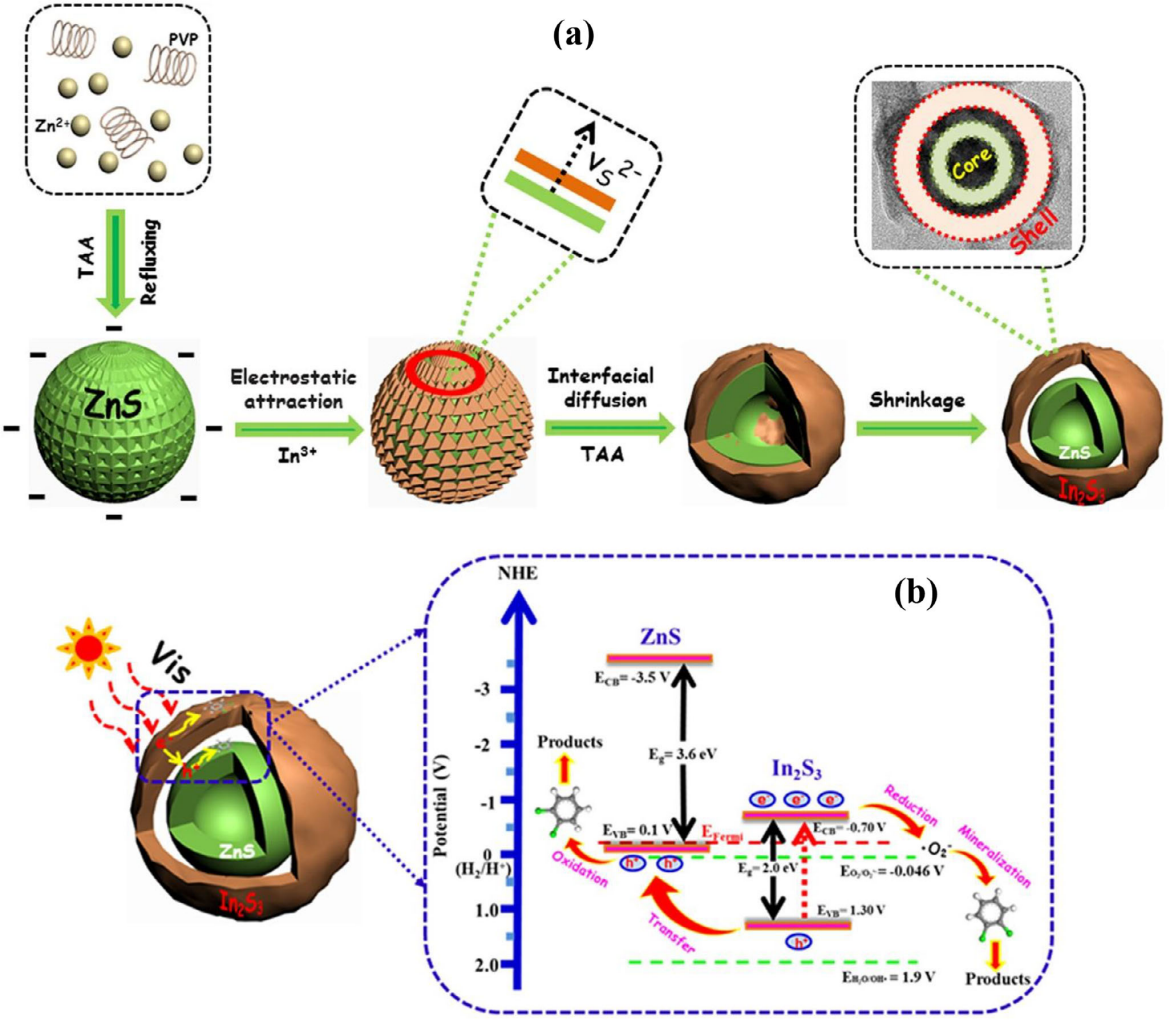
a) The schematic growth mechanism of hollow spheres with ZnS@In_2_S_3_ core@shell. b) Schematic of the ZnS@In_2_S_3_ core‐gaseous shell's o‐Dichlorobenzene degradation process.^[^ ^101^ ^]^ Copyright (2017) Springer Nature.

Brahmi et al.^[^ ^100^ ^]^ successfully combined perovskites and polymer by a quick, inexpensive, and environmentally friendly photopolymerization technique using a visible light emitting diode (LED) @405 nm. Medium viscosity and three readily available reactive functions make it trimethylolpropane tri acrylate (TMPTA), which was discovered to be the most suited monomer for the synthesis of the composites. The composites based on TMPTA monomer were also found to be stable in water. The newly produced materials were thoroughly described using SEM, TEM, EDX, and DRX which proved that perovskites and polymer composites could be successfully hybridized. According to studies using the AFM, DMA, and ATG technologies, the produced shaped materials also demonstrated great stiffness and high thermal stability. Compared to titanium dioxide (TiO_2_) the immobilized perovskites demonstrated enhanced photocatalytic activity under UV–vis light exposure. After only 30 min of UV irradiation, ≈94% of the chosen dye was eliminated from water when 2% Nd_0.9_TiO_3_/polymer was present and 95% when 2% LaTiO_3_/polymer was present. Liu et al.^[^ ^101^ ^]^ reported that an ion exchange process between ZnS and In^3+^ ions, a ZnS@In_2_S_3_ core@shell hollow composite is created, as shown in **Figure** **9**a. Its surface shape was identified with the help of a FESEM, TEM, XPS, and UV–vis DRS spectrophotometer. BET is also applied to evaluate prepared materials' pore volume and specific surface area. Because of their band alignment and unique design, the breakdown of gaseous o‐Dichlorobenzene by the core‐shell hollow nanospheres demonstrated increased catalytic activity as a photocatalyst as shown in Figure 9b. After 8 h of exposure to visible light (>400 nm), this material's o‐DCB degradation rate was 49%, which is mostly attributable to its effective charge separation ability.

Using numerical and experimental techniques, Nguyenet et al.^[^ ^102^ ^]^ examined the ZnO nanorods (NR)/CuO composite film's photocatalytic activity. The sample was initially produced on a glass substrate using sputtering and thermal annealing procedures. The ZnO NRs film was then hydrothermally produced on top of the CuO layer. The sample was then cleaned using an ultrasonic cleaner, ethanol, and deionized water. The sample's optical absorption spectra, surface morphology, and crystal phase were each examined using XRD, FESEM, and UV–vis spectrophotometry, respectively. Under the light of a Xenon lamp, the rate of RhB contamination removal acted as a measure of the materials' photocatalytic efficiency. Degradation efficiencies for the various samples were 99% in the presence of ZnO NRs/CuO composite films,78% in the case of ZnO NRs film, and 55% when using CuO film according to the results after 120 min. This work has shown that fusion of the ZnO and CuO materials might increase the possibility for deterioration of the ZnO material. After three cycle studies, the ZnO NRs/CuO composite film's photocatalyst still exhibits a high degradation rate toward RhB contamination. The photocatalyst made of ZnO NRs and CuO was very effective and economical.

Islam et al.^[^ ^103^ ^]^ reported that a sol–gel process was employed to produce nanoparticles from mesoporous titanium (TNPs) and nanocomposite from silica and titanium (STNC) with CdTe to form (CdTe/STNC), and examine its application as photocatalyst. The nanostructure of the CdTe‐based STNC catalyst was discovered using VPSEM. From TEM, they found that TNPs have diameters of 5.6 nm, STNC has 2.6 nm, and CdTe/STNC has 2.1 nm and displayed spherical morphologies. ATR‐FTIR may be used to analyse the bonding between functional groups, CdTe, titania, and silica. The XRD study's findings showed a high correlation between silica and CdTe species and the titania's brookite and anatase phases. The CdTe/STNC demonstrated excellent photocatalytic efficiency reached 95% greater than STNC (81%), as well as (58%) TNPs. Measurements suggest that the heterogeneous nano photocatalyst CdTe/STNC may be employed to remove organic pollutants from water waste.

Tripta et al.^[^ ^104^ ^]^ developed a chemical co‐precipitation was applied to generate CdO, NiFe_2_O_4_, and NiFe_2_O_4_@CdO nanocomposites to improve the photocatalyst's efficiency examining the FT‐IR, XRD, UV–vis, XPS, PL, and electrical properties of generated materials to identify their functional, vacancy defect, optical, structural, and morphological characteristics. A model pollutant called MB was employed to measure photocatalytic activity. Results from photocatalytic analysis indicate the nanocomposite exhibits good degradative activity and speeds up the process compared to nickel ferrite and cadmium oxide. It had good magnetic separation at room temperature and destroyed ≈91% of the MB dye. Uses for NiFe_2_O_4_@CdO nanocomposites in environmental remediation and contamination removal are possible.

Medha and Thaku^[^ ^105^ ^]^ reported that by grafting C_3_H_5_NOonto the polymer chainof chitosan and gelatinewhen there was (NH_4_)_2_S_2_O_8_as an initiator and CHCO_2_Has a cross‐linker, the composite hydrogel containing ZnONPs was created. This nanocomposite was further investigated and assessed using XRD, FTIR, and SEM. Elemental analysis could be used to determine the hydrogel's elemental makeup. The nanocomposite hydrogel significantly increased its photocatalytic activity for CR (90.8%) removal when exposed to sunshine. A promising photocatalyst has high efficiency for up to 4 cycles of CR dye removal from wastewater. The results offered a simple one‐pot technique for a natural polymer‐based nanocomposite hydrogel production in an aqueous solution with nanoparticle addition.

Manda et al.^[^ ^106^ ^]^ reported that a ZnO‐TiO_2_ (ZT) & ZnO‐TiO_2_‐reduced graphene oxide (ZT‐RGO) nanocomposites, and a pulse laser ablation approach were used in photocatalysis. Testing was done on the nano catalyst materials using SEM, AFM, TEM, FT‐IR, TGA, and UV–vis after they had been made. The developed materials were tested for their capacity to photodegrade the standard dye pollutant MB under UV light. To assess the impact of RGO loading with percentages 5, 10, and 20, the kind of nanocrystal structures produced, the increased thermal stability attained, and the effective removal of MB dye were employed. The results indicated that the completed nanocomposite can be created in 30 min and can remove 98.5% of the MB. ZT‐RGO, a 5% nanocomposite, was the most active in terms of photocatalysis. Li and Wang^[^ ^107^ ^]^reported that without the use of an organic solvent or surfactant, a straightforward one‐step homogeneous coprecipitation procedure was used to successfully create a nanostructured ZnO‐CuO composite. The materials were characterized using SEM, TEM, XRD, UV–vis, and XPS. Findings from the study showed that the ZnO‐CuO nanocomposite was decorated with CuO nanopatchesthat resembled leaves shapes ZnO microstructures have resembled flowers shapes, and that it had a hierarchical 3D form RhB was used to assess the ZnO‐CuO nanocomposite's photocatalytic activity. These results revealed that the prepared nanocomposite had strong photocatalytic efficiency. The RhB degradation was around 37% in presence of ZnO pure and 56% in presence of CuO pure photocatalysts in 120 min. RhB photodegradation in the ZnO‐CuO nanocomposite achieved 100% after 120 min of Xe light exposure.

**Figure 10:**
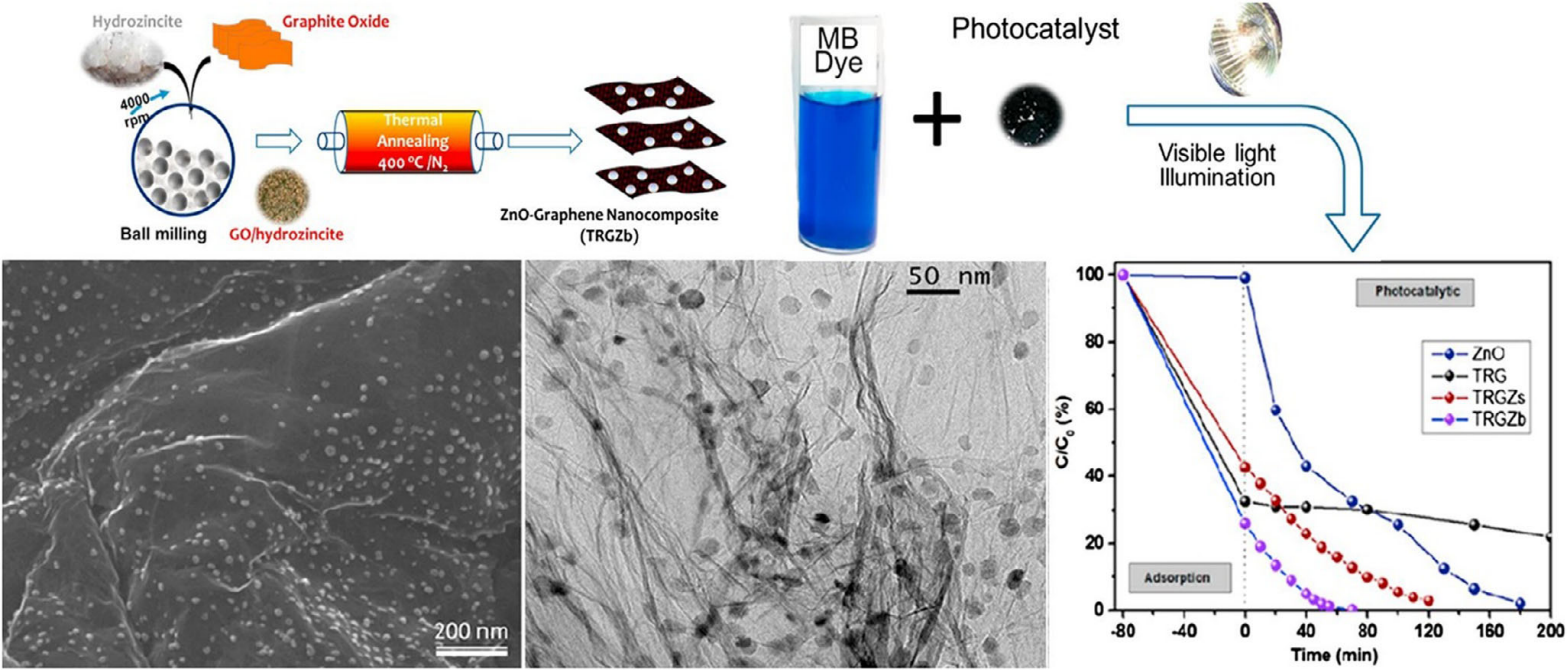
Formation of ZnO‐graphene nanocomposite. With permission from ref. [108] Copyright (2019) Elsevier.

Lonkar et al.^[^ ^108^ ^]^ developed a ZnO‐graphene nanocomposites were made using multistep solution‐based processes; however, these techniques produced a lot of liquid waste and were ineffectual. The researchers in this study were given a revolutionary solvent less method to create ZnO‐graphene nanocomposites. These materials were made by thermally annealing graphite oxide and hydrozincite. A quick ball milling step to produce materials with equally distributed ZnO nanoparticles with particle size 9 nm on thermally reduced graphene (TRG) surface as explained in **Figure** **10**. Analysis of the structure and morphology reveals that the nanocomposite produced by ball milling has a larger surface area and a smaller particle size of ZnO than that produced by the solution‐based hydrothermal method. The tests of the shape and structure were performed using SEM, TEM, TGA, XRD, FTIR, XPS, and BET. To study the photocatalytic activity for prepared MB dye selected as an example. They found that the high‐performance metal oxide‐graphene nanocomposites for photocatalytic applications can be synthesized quickly, safely, and scalable using this chemothermal method. Reaching to complete dye degradation (100%) in 70 min by TRGZb, 120 min by TRGZs and 180 min by ZnO.

**Figure 11:**
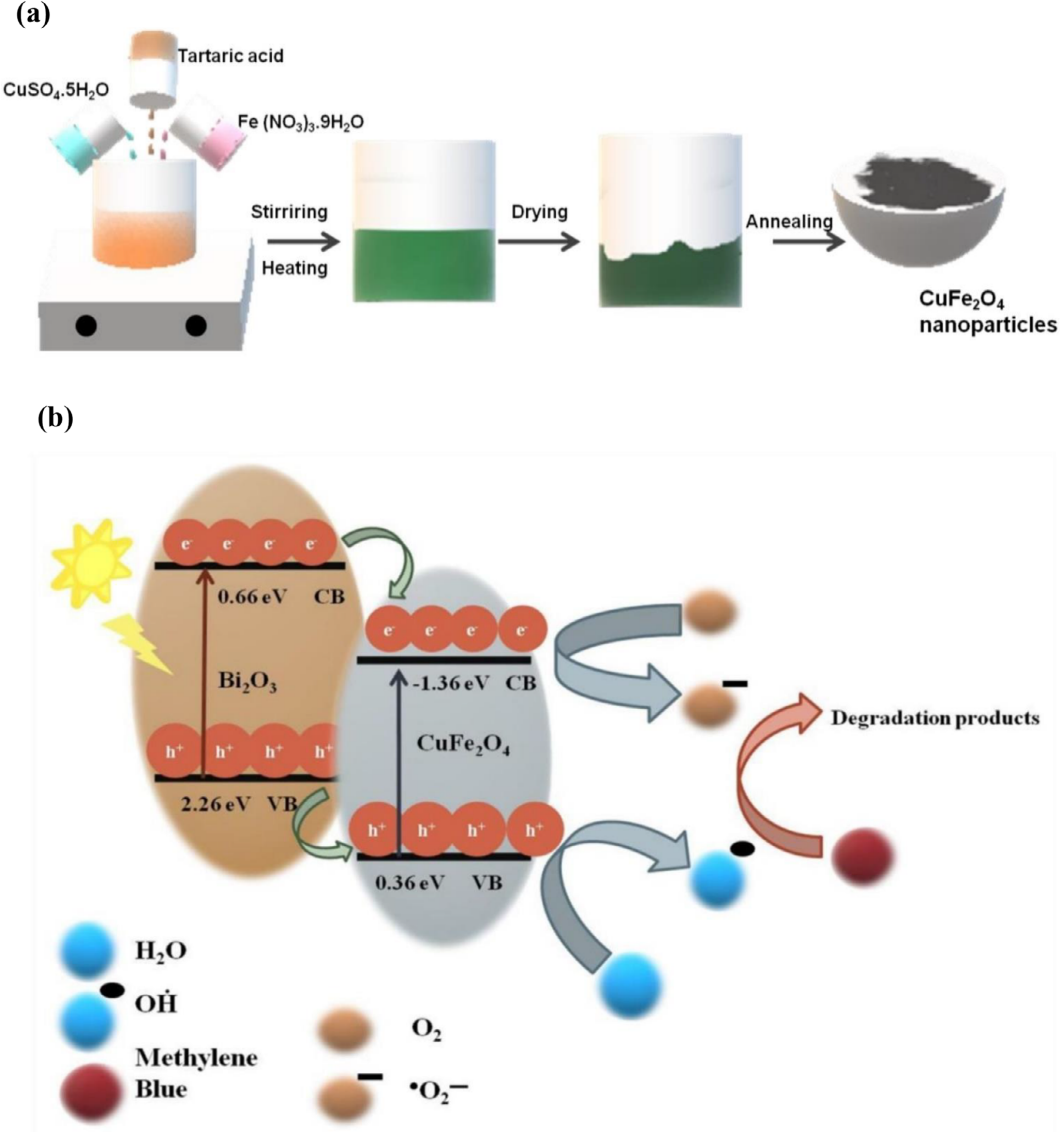
a) A schematic showing how to make nanocomposites of CuFe2O4 and Bi2O3, and b) CuFe2O4/Bi2O3 nanocomposite's suggested mechanism for photo catalytically degrading MB. With permission from ref. [109] Copyright (2019) Elsevier.

Munir et al.^[^ ^109^ ^]^ reported that by adopting a straightforward sonication approach to introduce Bi_2_O_3_ nanoparticles into the porous CuFe_2_O_4_ nanoparticles, a promising magnetic CuFe_2_O_4_/Bi_2_O_3_ nanocomposite was created, as shown in **Figure** **11**a. Many physicochemical characteristics of the generated materials, including the crystallite size, shape, and optical properties, were tested using XRD, FT‐IR, UV–vis, FESEM, and EDX. CuFe_2_O_4_/Bi_2_O_3_ nanocomposite may be proven to have high photo‐degradation performance toward the elimination of MB dye because of a decrease in recombination and enhanced separation of electron (e^‐^)‐hole (h^+^) pairs as can be seen in Figure 11b. It had excellent magnetic separation at ambient temperature and degraded ≈91% of the MB dye. Nanocomposites made of CuFe_2_O_4_ and Bi_2_O_3_ may be used to clean up the environment and remove impurities.

Gördük et al.^[^ ^110^ ^]^ developed a ZnO‐graphene nanocomposites using multistep solution‐based processes; however, these techniques produced a lot of liquid waste and were ineffectual. The in situ hydrothermal technique was used to create phthalocyanine (Pc)‐TiO_2_ nanocomposites (a). Utilizing PC composites devoid of metal and composed of Cu, Zn (II), Co (II), and Ni (II). FEG‐SEM, EDX, BET, FT‐IR, and UV‐DRS methods were chosen to examine the five different Pc‐TiO_2_ nanoparticles. From the UV–vis spectra, they assessed the photocatalytic efficiency for prepared materials. They found that these materials could be applied as photocatalysts to eliminate MB dye under vis‐light irradiation. High photocatalytic performance of Pc‐TiO_2_ photocatalysts proved that the prepared material breakdown MB totally in 100–130 min for full degradation (100%). According to studies on reusability, photocatalysts maintain 76% of their activity even after five usages.

A ZnO‐saponite nanocomposite prepared through a sol–gel process for environmental remediation and photocatalysis mechanisms was further applied as the source for photodegradation of Rhodamine B (RhB) as a model dye via visible light irradiation.^[^ ^111^ ^]^

The materials were characterized for their structure, morphology, and optical properties with the aid of different techniques such as X‐ray diffraction, SEM, FTIR, and photoluminescence. These results outline that this kind of nanocomposite is effective in incorporating semiconductors onto the support surface, forming a hexagonal structure with ZnO nanoparticles. The synthesized photocatalyst showed a more than 85% removal efficiency of the RhB dye in 270 min, and the order of kinetics came out to be pseudo‐first‐order. Alcohol played an important role in scavenging hydroxyl radicals. The nanocomposites were found to be stable after successive cycles, without showing toxicity, and thus became great candidates for the removal of polluting dye. The N‐CuO@Zeolite nanocomposite was synthesized using an environmentally friendly natural extract of the Camellia sinensis plant, which has been shown to effectively degrade hazardous dye pollutants, particularly AO and YD, in wastewater environmental treatment.

Embedding nitrogen and zeolite into the matrix of this nanocomposite resulted in enhancement of its photocatalytic properties with regard to negative zeta potential, lower bandgap energy, and higher surface area for adsorption and interaction with dye molecules. These characteristics were responsible for the efficient photodegradation of AO and YD dyes under solar radiation, hence indicating a great achievement in photocatalytic pollutant degradation. The synthesis route supports the possibility of using renewable resources in environmental detoxification and successful engineering of nanocomposites toward long‐term water‐purifying technologies. The optimum values of degradation efficiencies were obtained at pollutant concentrations of 2 and 6 mg L− 1, respectively, while the photocatalyst dosage used was 20 mg for both AO and YD.

The green modification of CuO through nitrogen doping and the introduction of zeolite frameworks indeed show great promise in applications related to sunlight‐driven photocatalysis, with no hazardous chemicals used during the synthesis process. No artificial light sources are involved in this. This nanocomposite exhibits outstanding stability and recyclability up to 10 cycles, representing a pragmatic solution for real‐life applications in terms of their applications with a view to reducing environmental pollution.^[^ ^112^ ^]^

Phamet al.^[^ ^113^ ^]^ explained the formation of the photo catalytically degradable polyethylene glycol (PEG)‐Fe_3_O_4_/ZnO magnetic nanocomposite utilising an ecologically friendly chemical synthesis technique using rambutan peel extract as a stabilising agent. The produced nanocomposites were studied using UV–vis, FT‐IR, XRD, and EDX methods. The (PEG)‐Fe_3_O_4_/ZnO magnetic nanocomposite's photocatalytic performance was tested by eliminating MB dye. As a result, almost 98% of MB was able to be removed by the photocatalyst in less than 90 min. Depicted an illustration of the photocatalytic degradation mechanism for MB.

Thakur et al.^[^ ^114^ ^]^ reported that a Gelatin‐Zr (IV) phosphate (GT/ZPNC) nanocomposite was made according to mixing inorganic Zr(IV) phosphate with gelatin gel (ZP) using the sol–gel method. The GT/ZPNC was examined using FT‐IR, XRD, TGA, XPS, and SEM techniques. GT/ZPNC effectively removed the dyes MB and fast FG from aqueous solutions. The studies showed that the GT/ZPNC nanocomposite had eliminated 89.91% from FG and 87.81%from MB after 5 h. Antibacterial treatment using GT/ZPNC against the E. coli bacterium was similarly successful. All the previous data are collected in **Table** **2**, which contains the Different synthesized nanocomposites used in the photocatalytic technique.

**Table 2:** Nanocomposites in photocatalytic.

Authors	Nano photocatalyst	Ligands/Metalloligands	Active sites	Synthesis methods	Characterization techniques	Contaminant	Light source	Results
Dadan et al.^[^ ^94^ ^]^	TiO_2_/ZnO/SiO_2_ (TZS) nanocomposite.	Zinc Oxide, and Titanium Dioxide	Silica matrix	Sol–gel‐hydrothermal method	XRD, SEM‐EDX, TEM, surface area measurements, and bandgap energy analysis.	Lead ions (Pb^2^⁺)	160‐watt mercury lamp	TZS synthesized with water‐ILs achieved a 99.98% removal of Pb (II) ions, outperforming the water‐synthesized TZS, which reached 95.49%
Romolini et al.^[^ ^95^ ^]^	Ag‐SiO_2_ Nanocomposite	3‐aminopropyltriethoxysilane (APTES)/ 3‐mercaptopropionic acid (MPA)	Silver nanoparticles (Ag NPs)/ Silica defects	Sol–gel method and then functionalized with a post‐synthesis process	TEM, FE‐SEM, EDX, and FT‐IR spectra	9‐anthracenecarboxylic acid (9ACA)	White light source (WL)/405 to 407 nm	Provides 60% photodegradation
Babyszko et al.^[^ ^96^ ^]^	SiO_2_/TiO_2_ photocatalysts	Silica (SiO_2_)	TiO_2_ (anatase and brookite phases)/ hydroxyl groups (‐OH)	Sol–gel method	XRD, SEM, FT‐IR, and UV–vis	MB	UV light with an intensity of 138 W m^−^^2^ in the range of 280–400 nm	SiO_2_(14.3%)/TiO_2_: 42.73% MB degradation under the same conditions.
Akash Kumar et al.^[^ ^97^ ^]^	ZnO/PANI/RGO ternary nanocomposite	Reduced Graphene Oxide (RGO)/ Polyaniline (PANI)	Zinc Oxide, Amino Groups (‐NH‐), Residual ‐OH, and ‐COOH	Physical Blending Method	FT‐IR, XRD, and UV–vis	MO	Sunlight	Methyl orange was utilized to assess the photocatalytic performance, achieving a 99% degradation rate within 50 min.
Aslam et al.^[^ ^98^ ^]^	Sm/SnO_2_‐SiO_2_	N‐dodecyl‐N,N‐dimethyl‐3‐ammonium‐1‐propanesulfonate (SB3‐12)	Samarium doping (Sm^3^⁺)/ SnO_2_ (Tin Oxide)/ SiO_2_ (Silica)	Combination of sol–gel synthesis and hydrothermal doping	EDX, TGA, TEM, XRD, FT‐IR, and XRD	MB	Sunlight	Percent degradation for SnO_2_‐SiO_2_ and Sm/SnO_2_‐SiO_2_ nanocomposites were found to be 71 and 78%
Al‐Rawashdeh et al.^[^ ^99^ ^]^	Graphene oxide (GO)/ZnO nanocomposites with embedded metal nanoparticles (Ag, Cu)	Graphene oxide (GO)	Silver, Zinc oxide, Hydroxyl (‐OH), epoxy (‐C‐O‐C), and carboxyl (‐COOH) groups	One‐Pot Microwave‐Assisted Method	FT‐IR, SEM, and XRD	MB	Sunlight using a PECCEL PEC‐l01 portable solar simulator (wavelength range: 300–1400 nm).	GO‐ZnO: 84% degradation of MB in 90 min under sunlight. GO‐ZnO‐Ag: 100% degradation of MB in 40 min under sunlight.
Brahmi et al.^[^ ^100^ ^]^	Combine perovskites and polymer (1% Nd_0.9_TiO_3_/polymer and 1% LaTiO_3_/polymerx)	Trimethylolpropane triacrylate (TMPTA)	Titanium (Ti) sites/ Oxygen vacancies/ Lanthanum (La) or Neodymium (Nd) sites/ Residual acrylate or hydroxyl groups	Photopolymerization	SEM, TEM, EDX, DRX, AFM, DMA, and ATG technologies	Acid Black dye	Omnicure Dynamic lamp, series 1000 lm (I0 = 250 mW cm^−2^, λ = 320–520 nm)	After only 30 min of UV irradiation, ≈94% of the chosen dye was eliminated from water when 2% Nd_0.9_TiO_3_/polymer was present and 95% when 2% LaTiO_3_/polymer was present.
Liu et al.^[^ ^101^ ^]^	ZnS@In_2_S_3_ core@shell hollow composite	Polyvinylpyrrolidone (PVP)	In_2_S_3_ (Indium Sulfide), and ZnS (Zinc Sulfide)	Ion‐exchange reaction	FESEM, TEM, XPS, BET, and UV–vis DRS spectrophotometer.	Gaseous o‐DCB	Visible‐light irradiation (λ>400 nm)	O‐Dichlorobenzene (o‐DCB) degrades at a rate of 49% after being exposed to visible light for 8 h.
Nguyen et al.^[^ ^102^ ^]^	ZnO NR/CuO composite film	–	Copper Oxide, and Zinc Oxide	Layer‐by‐Layer Deposition	XRD, FESEM, and UV–vis spectrophotometry	RhB	A 250 W Xenon lamp with wavelengths in the UV–vis spectrum	CuO film alone: 55% RhB degradation in 120 min. ZnO NRs alone: 78% RhB degradation in 120 min. ZnO NRs/CuO composite: 93% RhB degradation in 120 min.
Islam et al.^[^ ^103^ ^]^	CdTe/SiO_2_‐TiO_2_ Nanocomposite (CdTe/STNC)	Cetyltrimethylammonium Bromide (CTAB)	CdTe/ SiO_2_‐TiO_2_ Nanocomposite/ Hydroxyl (‐OH) groups	Sol–gel Method	VPSEM, TEM, ATR‐FTIR, and XRD	MB	A UV–vis lamp emitting at a wavelength of 254 nm	High photocatalytic activity was demonstrated by the CdTe/STNC, 95% greater than by STNC (81%), and TNPs (58%).
Tripta et al.^[^ ^104^ ^]^	NiFe_2_O_4_@CdO	Oleic Acid	NiFe_2_O_4_/ CdO/ Hydroxyl Radicals (•OH) and Photo‐Generated Holes (h⁺)	Chemical Co‐Precipitation Method	FT‐IR, XRD, UV–vis, XPS, PL, and electrical properties	MB	A 300 W OSRAM UV–vis lamp	The degradation percentage in the absence of any of the scavengers is 91% for MB and has excellent magnetic separation at room temperature.
Medha and Thaku^[^ ^105^ ^]^	CG‐g‐poly(AAm)‐ZnO Hydrogel	Maleic Acid/ Acrylamide (AAm)	ZnO nanoparticles/ ‐COOH, ‐NH_2_, and ‐OH	Chemical Co‐Precipitation and Graft Polymerization for Nanocomposite Hydrogel Formation	XRD, FTIR, and SEM	Congo Red Dye	Sunlight: 850 lx intensity	In sunlight, the nanocomposite hydrogel showed significant photocatalytic activity toward CR dye (90.8%).
Manda et al.^[^ ^106^ ^]^	ZnO‐TiO_2_ (ZT) & ZnO‐TiO_2_‐reduced graphene oxide (ZT‐RGO)	Reduced graphene oxide (rGO)	Titanium Dioxide, and Zinc oxide	One‐pot laser ablation	SEM, AFM, TEM, FT‐IR, TGA, and UV–vis	MB	A 15 W VL‐215.LC UV light source with a wavelength of 365 nm	The results indicated that the completed nanocomposite can be created in 30 min and can remove 98.5% of the MB. ZT‐RGO 5% nanocomposite was the most active in terms of photocatalysis
Li and Wang^[^ ^107^ ^]^	ZnO–CuO composite	–	Copper oxide, and Zinc oxide	One‐Step Homogeneous Co‐Precipitation Method	SEM, TEM, XRD, UV–vis, and XPS	RhB	Simulated sunlight provided by a 300 W Xenon lamp	The RhB degradation is around 37% for pure ZnO and 56% for pure CuO photocatalysts at 120 min, respectively. After 120 min of exposure to the Xe lamp, the photodegradation of RhB in ZnO‐CuO nanocomposite had reached over 100%.
Pillai et al.^[^ ^108^ ^]^	ZnO‐graphene nanocomposites	Graphene	Zinc oxide/ Graphene (TRG)/ Residual ‐OH, ‐COOH, and epoxy groups	Mechanothermal (Ball Milling) Method	SEM, TEM, TGA, XRD, FTIR, XPS, and BET	MB	A 400 W metal halide lamp providing visible light	Reaching to complete dye degradation (100%) in 70 min by TRGZb, 120 min by TRGZs and 180 min by ZnO.
Rasheed et al.^[^ ^109^ ^]^	CuFe_2_O_4_/Bi_2_O_3_ nanocomposite	Tartaric acid	CuFe_2_O_4_ (Copper Ferrite), and Bi_2_O_3_ (Bismuth Oxide)	Sol–Gel Method/ CuFe_2_O_4_/Bi_2_O_3_ Nanocomposite Formation: Ultrasonication‐Assisted Method	using XRD, FT‐IR, UV–vis, FESEM, and EDX	MB	A sodium lamp emitting visible light	It had excellent magnetic separation at ambient temperature and degraded ≈91% of the MB dye.
Gorduk et al.^[^ ^110^ ^]^	Phthalocyanine–TiO_2_ Nanocomposites (Pc–TiO_2_)	Phthalocyanines (Pc) derivatives	TiO_2_ (Titanium Dioxide), and Phthalocyanine (Pc)	Hydrothermal in situ Method	FEG‐SEM, EDX, BET, FT‐IR, and UV‐DRS	MB	A 250 W visible‐light lamp	Pc‐TiO_2_ photocatalysts have strong photocatalytic activity, which accelerates the degradation of MB to 100% in 100–130 min. Studies on reusability show that even after five uses, photocatalysts retain 76% of their activity.
Dihêgo et al.^[^ ^111^ ^]^	ZnO‐saponite nanocomposite	Cetyltrimethylammonium Bromide (CTAB)	ZnO (Zinc Oxide)/ Saponite Clay	Two‐step process combining surfactant‐assisted modification and thermal synthesis	X‐ray diffraction, SEM, FTIR, and photoluminescence	RhB	A 160 W mercury vapor lamp emitting UV–vis light.	Achieved over 85% removal of RhB dye after 270 min.
Vipin et al.^[^ ^112^ ^]^	N‐doped CuO@Zeolite Nanocomposite (N‐CuO@Zeolite)	Zeolite framework	Copper Oxide/ Nitrogen doping	Green synthesis with co‐precipitation	Zeta potential measurements, bandgap energy analysis, and surface area measurements.	Auramine‐O (AO), and yellow color textile industrial dye (YD)	Solar radiation	The N‐CuO@Zeolite nanocomposite demonstrated higher degradation efficiencies, achieving 95% for AO and 92% for YD.
PHAM et al.^[^ ^113^ ^]^	PEG‐Fe_3_O_4_/ZnO nanocomposite.	PEG (Polyethylene Glycol)	Fe_3_O_4_ (Iron Oxide), ZnO (Zinc Oxide), and Hydroxyl (‐OH) groups from PEG and ZnO aid in pollutant adsorption	Green Sonochemical Synthesis	UV–vis, FT‐IR, XRD, and EDX	MB	UV Light from a 500 W source.	Achieved 98% degradation of methylene blue within 90 min under UV light.
Thakur et al.^[^ ^114^ ^]^	Gelatin‐Zr(IV) Phosphate Nanocomposite (GT/ZPNC)	Gelatin Matrix	Zirconium(IV) Phosphate/ Amine Groups (‐NH_2_)/ Carboxyl Groups (‐COOH)	Sol–gel method	FT‐IR, XRD, TGA, XPS, and SEM techniques	E. coli, fast green (FG), and MB	Solar light	Methylene Blue (MB): 87.81% degradation within 5 h. Fast Green (FG): 89.91% degradation within 5 h.

Nanocomposites are very useful in cleaning wastewater, but their performance changes depending on the type of pollutant, such as (dyes, heavy metals, or nutrients), that largely influenced by their composition, structure, and surface properties as revealed through extensive characterization in research. Metal oxide‐based photocatalysts such as TiO_2_, MnO_2_/CuO/Fe_2_O_3_, and ZnO are prominent for dye degradation, benefiting from high surface area and suitable bandgap energies (≈1.3–3.2 eV) that enable effective activation under UV or visible light. For example, ZnO nanocomposites with hexagonal crystalline phases showed superior photocatalytic activity compared to TiO_2_ and SnO_2_ in degrading dyes like crystal violet under solar irradiation, partly due to their morphology and light absorption properties as shown in **Table** **3**.^[^ ^115^ ^]^

**Table 3:** Efficiency Against Different Pollutants.

Pollutant type	Much effective nanocomposite types	Efficiency factors and variables
Dyes	Metal oxide‐based photocatalysts: TiO_2_, MnO_2_/CuO/Fe_2_O_3_, ZnO	High surface area, suitable bandgap energy (e.g., ≈1.3–3.2 eV), light absorption properties, particle size, crystallinity, morphology.^[^ ^119^ ^]^ Slower kinetics observed for some dyes requiring longer irradiation or co‐catalysts.^[^ ^120^ ^]^
Heavy Metals	Carbon‐based (e.g., functionalized MWCNT/polymer composites), metal oxides, biopolymeric nanocomposites	Presence of oxygen‐containing functional groups (carboxyl, hydroxyl) enhances adsorption via electrostatic interaction and complexation; porosity and surface roughness key; composition tuning affects selectivity for metals like Co^2+^, Pb^2+^, As^3+^.^[^ ^121^, ^122^, ^123^ ^]^ Less reusability when poisoning or surface saturation occurs.
Nutrients (N, P compounds)	Less studied; some metal oxide nanocomposites for phosphate adsorption, bio‐nanocomposites for nitrogen removal	Efficiency depends on ion‐exchange capacity, surface charge, and composite surface area; competitive adsorption with other ions possible; fewer robust studies showing complete nutrient removal.^[^ ^124^ ^]^

Iron oxide‐based nanocomposites also demonstrated notable dye degradation efficiency of up to 94% for rhodamine B under visible light, reflecting the impact of elemental composition and bandgap tuning on photocatalytic performance. When it comes to removing heavy metals, magnetic nanocomposites like ZnFe_2_O_4_ and CoFe_2_O_4_ are great because they can attract metals and be pulled out of water using magnets, staying effective through ≈4–6 uses. Carbon‐based materials with special chemical groups (like –OH and –COOH) also help remove metals such as lead and cobalt by attracting them through charge interactions, but they don't last as long. Removing nutrients like nitrates and phosphates is harder; only a few nanocomposites, mostly biopolymer or metal oxide types, can do this moderately well, and their success is often reduced by other substances in real wastewater.

Several key factors influence how effectively nanocomposites remove pollutants from wastewater. These include their chemical makeup, where adding noble or rare‐earth metals can boost the separation of charges and the formation of reactive oxygen species that help break down contaminants.^[^ ^116^ ^]^ The structure and shape of nanocomposites, like having rough, porous surfaces and well‐controlled particle sizes, which improve their ability to adsorb pollutants and speed up reactions. A larger surface area offers more active sites for treatment, and optical properties such as a tuned bandgap help match the nanocomposite's light absorption with visible or UV light for efficient photocatalysis.^[^ ^117^ ^]^ However, several challenges remain. Many nanocomposites degrade slowly, especially in treating dyes and nutrients, requiring long exposure times and slowing down treatment. Their surfaces can also become fouled or poisoned, reducing their lifespan and requiring regeneration. There's also a risk of nanoparticle leaching which could harm the environment. Furthermore, it's still difficult to mass‐produce consistent, high‐quality nanocomposites. While metal oxide nanocomposites are effective against dyes, and magnetic or carbon‐based types are good for heavy metal removal, treating nutrients still needs better solutions. Future success depends on carefully adjusting the synthesis methods and surface properties to improve performance and stability on a large scale.^[^ ^118^ ^]^

## 5 Challenges Faced by Nanocomposites in Wastewater Treatment

Nanocomposites possess exceptional adsorption capabilities and catalytic efficiency, positioning them as strong contenders for next‐generation wastewater treatment solutions. Despite their promising performance in controlled laboratory settings, transitioning these materials to large‐scale, real‐world applications presents numerous practical and technological obstacles. The key limitations are outlined below, with insights drawn from recent scientific studies and on‐site implementation data.

- Catalyst Recovery and Reusability: Nanocomposite catalysts often exhibit excellent initial catalytic performance, but their durability across multiple reuse cycles can be inconsistent. For instance, magnetic nanocomposites may sustain high conversion rates (over 90%) for ≈4–5 cycles before a gradual decline, typically caused by surface contamination or structural degradation. However, their reusability is enhanced by the ease of separation using external magnetic fields, which simplifies recovery. Nevertheless, maintaining both high performance and operational simplicity remains essential for successful real‐world implementation.^[^ ^125^, ^126^ ^]^
- Environmental Impact: A major concern with nanocomposite use in wastewater treatment is the potential leaching of nanoparticles into the water, either during their synthesis, operation, or disposal.^[^ ^127^ ^]^ This leaching poses environmental and health risks due to the possible toxicity of nanomaterials and the uncertainty surrounding their long‐term effects. To address this, strategies such as surface functionalization with biopolymers or immobilization methods are employed to minimize particle release and aggregation. However, these approaches must be carefully tested across various types of wastewater to ensure their effectiveness and safety.^[^ ^128^ ^]^
- Long Term Stability: Nanocomposites often experience reduced efficiency over extended use, as factors like particle agglomeration, surface passivation, or chemical degradation impact their long‐term performance.^[^ ^122^ ^]^ To enhance durability, integrating biopolymers or forming hybrid structures has shown promise by improving structural integrity and resistance to wear. However, these stabilization strategies must be fine‐tuned to suit the diverse chemical compositions found in different wastewater systems for consistent and reliable performance.^[^ ^8^ ^]^
- Cost‐Effective Synthesis: Developing nanocomposites through traditional methods can be expensive and environmentally taxing, limiting their practicality for large‐scale wastewater treatment. In response, greener synthesis techniques, such as using plant‐based extracts and simplified one‐step processes, that are being explored to cut costs and reduce ecological impact.^[^ ^129^ ^]^ Despite these advancements, many nanocomposites remain costlier than conventional materials, posing a significant barrier to widespread use, particularly in low‐resource settings.
- Production Scalability: Scaling up nanocomposite production from laboratory to industrial levels presents significant hurdles, particularly in preserving uniformity, dispersion quality, and functional efficiency of the materials.^[^ ^130^ ^]^ Initiatives like the EU's NANOLEAP project have demonstrated that small process modifications can support scalability for both small and large manufacturers. However, broad commercial implementation is still constrained by high costs and the need for specialized technical expertise.
- Reactor Design for Large‐Scale Application: To use nanocomposites in real‐world water treatment, we need special reactors that match their properties. For example, membrane systems or setups that allow the catalysts to be easily removed and reused. Some advanced reactors, like continuous flow and membrane bioreactors, have been designed to work with nanocomposites and show good results in removing pollutants. However, they still need more testing to prove they work well on a larger, industrial scale.^[^ ^131^ ^]^
- Knowledge Gaps: There is still much to learn about the long‐term effects of nanomaterials on ecosystems and human health. Comprehensive research is needed to fill these knowledge gaps.^[^ ^122^ ^]^
- Energy Consumption: Some nanomaterial‐based wastewater treatment processes may require significant energy input, making them less sustainable in energy efficiency.^[^ ^132^ ^]^
- Optical properties: The optical properties of nanomaterials, such as their absorption spectra and bandgap energies, play a critical role in photocatalysis. Tuning these properties to match the target reaction and light source can be challenging.^[^ ^133^ ^]^
- Regulatory and safety considerations: As nanomaterials become more prevalent in photocatalytic applications, regulatory bodies are becoming more concerned about their safety and environmental impact. Complying with regulations and ensuring nanomaterials' safe handling and disposal is crucial.^[^ ^127^ ^]^

Addressing these challenges requires continued research and development efforts and collaboration between scientists, engineers, policymakers, and the public to ensure the safe and effective application of nanomaterials in wastewater treatment.

## 6 Conclusions and Recommendations for Future Work

A major challenge to living organisms and all of humanity is the lack of clean water and the difficulties in accessing it. Currently, the proportion of clean water worldwide is 2% of all available clean water. The reason for the decrease in the percentage of clean water and the increase in the percentage of polluted water is due to human activities. This problem prompted scientists to spend a lot of time and effort to find new ways and methods to solve this problem that threatens the world. After much research, they found that the remarkable properties of nanomaterials and their integration with existing technologies are a bright future for the current wastewater treatment revolution.

Despite their accomplishment, the researchers persisted with their work until they discovered even more remarkable qualities by mixing different nanomaterials with one another and astounding outcomes in the removal of dyes and other contaminants from water. These substances are referred to as nanocomposites. The high surface energy of these materials causes this characteristic development.

Some dyes, including methylene blue, Congo red, direct blue 14, sunset yellow, aniline blue, and others, showed a maximum clearance between 83 and 100%. After 120 min, more than 90% of Cr (VI) can be eliminated.

On the other hand, scientists have also been interested in creating photocatalysis materials that can help solve the water pollution problem. Additionally, the occurrence of this activity requires the presence of light and oxygen. Among their ongoing research, experts have come to the view that materials' photocatalytic properties are increased and developed by nanocomposites, which help clean up wastewater. Methyl orange has a breakdown rate of 99% in 50 min. When exposed to sunshine, the nanocomposite hydrogel significantly photocatalyzed Congo red (90.8%).

These results show the ability of nanocomposites to eliminate most or all the pollutants and dyes present in the water by different methods. The VOS viewer tool was used to perform a bibliometric analysis to demonstrate the trend in wastewater treatment using nanocomposites in accordance with author cooperation, country citation, journal bibliographic coupling, authors' keywords, and index keywords.

In the future, using nanocomposites for wastewater treatment is expected to improve in many smart and helpful ways. One big step forward is using magnetic nanocomposites, which can be easily removed from the water after treatment by using magnets. This makes the cleaning process cheaper, saves energy, and prevents leftover materials from staying in the water.

- Another important method is using Z‐scheme photocatalysts, which are special materials that work better under sunlight. These materials help separate electric charges more effectively and keep strong chemical power, which is useful for breaking down hard‐to‐remove pollutants and new harmful substances in water.
- Even better results can be achieved by combining nanomaterials with advanced oxidation processes (AOPs), like mixing photocatalysis with ozonation or Fenton reactions. These combinations make it possible to clean many types of dirty water, even when the water has different chemicals or pH levels. This makes the system more flexible and powerful.
- Computer simulations and artificial intelligence (AI) will also help researchers discover better nanocomposites faster. These tools can predict how a material will behave and help design new ones without wasting time or materials in the lab.
- To move from the lab to real‐world use, pilot‐scale studies are very important. These tests check if the nanocomposites work well in large systems, stay stable over time, and are easy to use in factories or water treatment plants.
- Finally, scientists must pay close attention to nanotoxicity, which is the possible harm that nanoparticles can cause to people or the environment. Future studies need to test how safe these materials are, find better ways to detect them in water, and follow safety rules to avoid any health risks.

The future of nanocomposites in water treatment depends on smarter materials, better system combinations, computer help, large‐scale testing, and making sure everything is safe. All of these steps together will help create cleaner, safer, and more efficient water treatment systems.

## Conflict of Interest

The authors declare no conflict of interest.
